# Supplementary material for: Minimal versus specialist equipment in the delivery of pulmonary rehabilitation: protocol for a non-inferiority randomised controlled trial
Source: BMJ Open. 2021 Oct 18;11(10):e047524. doi: 10.1136/bmjopen-2020-047524 (PMC8524266; doi:10.1136/bmjopen-2020-047524)
Supplement: Supplementary data [file bmjopen-2020-047524supp001.pdf]

BMJ Publishing Group Limited (BMJ) disclaims all liability and responsibility arising from any reliance placed on this supplemental material which has been supplied by the author(s)

Minimal versus specialist equipment in the delivery of pulmonary rehabilitation : Protocol for a non-inferiority randomised controlled trial

Online supplement

Consent: Informed consent form

Nolan CM, *et al. BMJ Open* 2021; 11:e047524. doi: 10.1136/bmjopen-2020-047524

PARTICIPANT CONSENT FORM

MInimal versus SpecialisT Equipment in the delivery of pulmonary Rehabilitation (MISTER): a randomised controlled trial

IRAS Reference: 241564

Name of Researcher: ....

Participant study ID number: ....

Please initial the boxes

1. I confirm that I have read and understand the participant information leaflet (version **4** dated **02/02/2021**) for the above study. I have had the opportunity to consider the information, ask questions and have had these answered satisfactorily. ☐

2. I understand that my participation is voluntary and that I am free to withdraw at any time without giving any reason, although data collected until that point may be used for research purposes with my agreement, without my medical care or legal rights being affected. ☐

3. I understand that sections of my medical notes relating to the study may be looked at by regulatory bodies conducting inspections which may include members of staff within the NHS Trust who are outside of the immediate clinical and research teams. I give permission for these individuals to have access to my records. ☐

4. a) I agree that the information held and maintained by NHS Digital and other NHS bodies will be used to provide information about my healthcare use, and in the case of my death, information about this. ☐

   b) I consent to share identifiable details (name, NHS number, date of birth, post-code) with NHS Digital. ☐

5. I consent to allowing my postcode to be used to calculate the Multiple Deprivation Index. ☐

6. I agree to my GP being informed of my participation in the study. ☐

7. I agree to take part in the above study. ☐

8. I wish to receive feedback on the study results when available. ☐

Name of Patient	Date	Signature
Name of Person taking consent (If different from researcher)	Date	Signature
Researcher	Date	Signature

When completed: 1 for patient; 1 (original) for researcher site file; 1 to be kept in medical notes

Nolan CM, *et al. BMJ Open* 2021; 11:e047524. doi: 10.1136/bmjopen-2020-047524

Study intervention

Home-exercise manual and study standard operating procedures: These can be provided on application to the Royal Brompton and Harefield Hospitals.

Education component of pulmonary rehabilitation

A multidisciplinary team, including physiotherapists, psychologists, dieticians, nurses, doctors, occupational therapists, dieticians, social workers, speech and language therapists and expert patients, delivered the education sessions. The will aim to develop patients' understanding and holistic management of their disease, and topics included pacing strategies, breathing control exercises, physical activity and exercise, medication use, diet, smoking cessation, management of anxiety and low mood, managing infections through early recognition, rescue medication, appropriate general practice/hospital presentation and loving relationships. The sessions will take place twice per week for 45 minutes and patients will receive a booklet of the topics covered in these sessions.

Safety measures during PR-min and PR-gym rehabilitation programmes

Safety measures for both types of programme:

- The programme shall be delivered at a suitable time and in easily accessible buildings and locations with adequate parking, good transport links and suitable for people with disabilities.
- A minimum of two members of staff are required to supervise group exercise classes and education sessions – one of whom must be a registered allied health professional.
- There will be a minimum of two members of staff supervising group exercise classes and education sessions in community settings. There is an absolute minimum of one staff to eight people for supervised group exercise. However, the ratio may increase to one staff to four people depending on the complexity and severity of the medical problems, and will be at the judgement of the responsible member of the senior clinical staff
- There should be no more than two ambulatory or long-term oxygen users in any one exercise class.
- Prior to each session a risk and suitability assessment of the venue must be undertaken.

- Unwell patients are managed using the emergency care protocol. In short, if urgent help is required in a community setting, call an ambulance, and in a hospital setting, call the crash team.
- Contraindications to exercise shall be screened at the pulmonary rehabilitation assessmen.
- Patients shall stop exercising if $Sp0_2$ <80% and if they feel unwell e.g. chest pain, nausea or if a member of staff feels it is necessary.
- The following monitoring equipment is required: pulse oximeters, blood pressure monitor
- The following emergency equipment is required:
    - Community-based programmes: portable defibrillator and bag-valve mask
    - Hospital-based programmes: crash trolley
    - Portable oxygen, tubing, face-mask
    - Glucose kit
    - Bio-hazard spill kit
    - First aid kit

Study outcome measures

Spirometry: Visit 1 and visit 3

All participants will undergo spirometry performing the Forced Vital Capacity (FVC) / Forced Expiratory Volume in one second (FEV_1) manoeuvre as described in Appendix 1, using an EasyOne™ diagnostic spirometer. As a minimum, three technically acceptable tests must be performed and must meet the Association of Respiratory Technology and Physiology reproducibility criteria.[11]. Results obtained will include FVC, FEV_1 and the FEV_1/FVC ratio. Percent predicted reference equations from the European Respiratory Society/European Steel and Coal Society were used.[12]

Frailty: Visit 1, visit 2 and visit 3

All participants will perform the Short Physical Performance Battery (SPPB) which is a simple test of lower limb functional performance and a marker of frailty.[13] It comprises an assessment of standing balance, usual walking speed

and ability to stand from a chair. The SPPB is scored out of 12 with a higher score indicating better functional performance. A score of <10 indicates a frail status and this cut-off will be used in the randomisation procedure.

Exercise capacity: Visit 1, visit 2 and visit 3

The ISW is an incremental, externally paced, field walking test that involves participants walking around a 10 metre course in time to a series of progressively faster beeps played from a CD player. At visit 1**,** two tests (a practice test and then the formal test) will be undertaken with at least 30 minutes rest between tests.[14] This is in accordance with international technical standards. Measures of breathlessness, oxygen saturation levels and heart rate will be recorded before and after the test. The distance completed in metres will also be recorded.

Dyspnoea: Visit 1, visit 2 and visit 3

Dyspnoea will be measured using the CRQ-D.[15] The dyspnoea domain allows the patients to choose five activities that have been limited by shortness of breath in the past two weeks.

Health-related quality of life: Visit 1, visit 2 and visit 3

Health-related quality of life will be measured using the CRQ.[15] This 20 item questionnaire, is responsive to PR, and contains four domains; dyspnea (described above), fatigue, emotional function and mastery, and a total score. Each item is scored on a seven point Likert scale, with a lower score indicating a higher symptom burden.

Muscle strength: Visit 1, visit 2 and visit 3

Isometric quadriceps maximal voluntary contraction of the dominant leg will be measured using specially designed chair and strain gauge. This test involves the participant pushing against an ankle strap with the knee positioned at 90^0.[16] A warm-up and six efforts will be performed.

Patient satisfaction: Visit 2

To measure patient satisfaction, participants will rate their response to the following question *"How do you feel your overall condition has changed after rehabilitation?"* on a five point Global Rating of Change Questionnaire. The scale ranges from *"1: I feel much better"* to *"5: I feel much worse".*

Health economic evaluation: Visit 1, visit 2 and visit 3

- The Modified Client Service Receipt Inventory questionnaire will be used to record information on health care resource, medicine and equipment use, informal care provided by family members, time off work and costs borne by the patient and family relating to the chronic lung disease.
- The EQ5D5L is a generic measure of health status that comprises a visual analogue scale and five-item questionnaire with the following domains: mobility; self-care; usual activities; pain/discomfort and anxiety/depression.[17]
- After visit 3, data will be obtained from NHS Digital regarding health resource utilization between visit 1 and visit 3. This will include information on elective and non-elective hospital admissions (e.g. reason for admission; type of ward; treatment received; length of stay (including date of admission and discharge); location of discharge) as well as outpatient healthcare contacts (e.g. type of appointment, number of contacts, treatment received) and mortality (e.g. date of death, reason for death, place of death).

Safety and trial process evaluation: Visit 1 and visit 2

- Safety will be assessed in real-time using adverse event reporting.
- PR uptake, adherence and completion will be assessed objectively through PR attendance registers and training records. Reasons for non-completion of PR will be assessed objectively through PR records.
