# Supplementary material for: Minimal versus specialist equipment in the delivery of pulmonary rehabilitation: protocol for a non-inferiority randomised controlled trial
Source: BMJ Open. 2021 Oct 18;11(10):e047524. doi: 10.1136/bmjopen-2020-047524 (PMC8524266; doi:10.1136/bmjopen-2020-047524)
Supplement: Author's manuscript [file bmjopen-2020-047524.draft_revisions.pdf]

BMJ Open is committed to open peer review. As part of this commitment we make the peer review history of every article we publish publicly available.

When an article is published we post the peer reviewers' comments and the authors' responses online. We also post the versions of the paper that were used during peer review. These are the versions that the peer review comments apply to.

The versions of the paper that follow are the versions that were submitted during the peer review process. They are not the versions of record or the final published versions. They should not be cited or distributed as the published version of this manuscript.

BMJ Open is an open access journal and the full, final, typeset and author-corrected version of record of the manuscript is available on our site with no access controls, subscription charges or pay-per-view fees (http://bmjopen.bmj.com).

If you have any questions on BMJ Open's open peer review process please email info.bmjopen@bmj.com

BMJ Open

MInimal versus SpecialisT Equipment in the delivery of pulmonary Rehabilitation (MISTER): a non-inferiority randomised controlled trial

Journal:	*BMJ Open*
Manuscript ID	bmjopen-2020-047524
Article Type:	Protocol
Date Submitted by the Author:	01-Dec-2020
Complete List of Authors:	Nolan, Claire; Royal Brompton and Harefield NHS Foundation Trust, Harefield Respiratory Research Group; Imperial College London, National Heart and Lung Institute<br>Walsh, Jessica; Royal Brompton and Harefield NHS Foundation Trust, Harefield Respiratory Research Group<br>Patel, Suhani; Royal Brompton and Harefield NHS Foundation Trust, Harefield Respiratory Research Group; Imperial College London, National Heart and Lung Institute<br>Barker, Ruth; Royal Brompton and Harefield NHS Foundation Trust, Harefield Respiratory Research Group; Imperial College London, National Heart and Lung Institute<br>Polgar, Oliver; Royal Brompton and Harefield NHS Foundation Trust, Harefield Respiratory Research Group<br>Maddocks, Matthew; King's College London, Cicely Saunders Institute, Division of Palliative Care, Policy & Rehabilitation<br>Gao, Wei; King's College London, Cicely Saunders Institute, Division of Palliative Care, Policy & Rehabilitation<br>Wilson, Rebecca; King's College London, Cicely Saunders Institute, Division of Palliative Care, Policy & Rehabilitation<br>Man, William; Imperial College London, National Heart and Lung Institute; Royal Brompton and Harefield NHS Foundation Trust, Harefield Respiratory Research Group
Keywords:	REHABILITATION MEDICINE, Chronic airways disease < THORACIC MEDICINE, Emphysema < THORACIC MEDICINE, Interstitial lung disease < THORACIC MEDICINE

SCHOLARONE™
Manuscripts

I, the Submitting Author has the right to grant and does grant on behalf of all authors of the Work (as defined in the below author licence), an exclusive licence and/or a non-exclusive licence for contributions from authors who are: i) UK Crown employees; ii) where BMJ has agreed a CC-BY licence shall apply, and/or iii) in accordance with the terms applicable for US Federal Government officers or employees acting as part of their official duties; on a worldwide, perpetual, irrevocable, royalty-free basis to BMJ Publishing Group Ltd ("BMJ") its licensees and where the relevant Journal is co-owned by BMJ to the co-owners of the Journal, to publish the Work in this journal and any other BMJ products and to exploit all rights, as set out in our licence.

The Submitting Author accepts and understands that any supply made under these terms is made by BMJ to the Submitting Author unless you are acting as an employee on behalf of your employer or a postgraduate student of an affiliated institution which is paying any applicable article publishing charge ("APC") for Open Access articles. Where the Submitting Author wishes to make the Work available on an Open Access basis (and intends to pay the relevant APC), the terms of reuse of such Open Access shall be governed by a Creative Commons licence – details of these licences and which Creative Commons licence will apply to this Work are set out in our licence referred to above.

Other than as permitted in any relevant BMJ Author's Self Archiving Policies, I confirm this Work has not been accepted for publication elsewhere, is not being considered for publication elsewhere and does not duplicate material already published. I confirm all authors consent to publication of this Work and authorise the granting of this licence.

For peer review only

MInimal versus SpecialisT Equipment in the delivery of pulmonary Rehabilitation (MISTER): a non-inferiority randomised controlled trial

Dr Claire M. Nolan PhD [1,2] c.nolan@rbht.nhs.uk

Mrs Jessica A Walsh [1] j.walsh@rbht.nhs.uk

Ms. Suhani Patel [1] s.patel1@rbht.nhs.uk

Ms Ruth E. Barker [1,2] r.barker2@rbht.nhs.uk

Mr Oliver Polgar [1] o.polgar@rbht.nhs.uk

Dr Matthew Maddocks PhD [3] matthew.maddocks@kcl.ac.uk

Dr Wei Gao PhD [3] wei.gao@kcl.ac.uk

Dr Rebecca Wilson PhD [3] rebecca.wilson@kcl.ac.uk

Dr William D-C. Man PhD [1,2,4] w.man@rbht.nhs.uk

Affiliations:

1. Harefield Respiratory Research Group, Royal Brompton and Harefield NHS Foundation Trust, UK
2. National Heart and Lung Institute, Imperial College London, UK
3. King's College London, Cicely Saunders Institute, Division of Palliative Care, Policy & Rehabilitation, London, UK
4. Harefield Pulmonary Rehabilitation Unit, Royal Brompton and Harefield NHS Foundation Trust, UK

Corresponding author: Dr Claire Nolan, Senior Research Physiotherapist, Royal Brompton and Harefield NHS Foundation Trust, Harefield Hospital, Middlesex, UB9 6JH, United Kingdom, Tel: +44 (0)1895 823737, Fax: +44 (0)1895 828889, Email: c.nolan@rbht.nhs.uk

For peer review only - http://bmjopen.bmj.com/site/about/guidelines.xhtml

Abstract

Introduction: Pulmonary rehabilitation (PR), an exercise and education programme for people with chronic lung disease, aims to improve exercise capacity, breathlessness and quality of life. Most evidence to support PR is from trials that utilise specialist exercise equipment e.g. treadmills (PR-gym). In contrast, a significant proportion of programmes do not have access to specialist equipment with training completed with minimal exercise equipment (PR-min). There is a paucity of robust literature examining the efficacy of supervised, centre-based PR-min. We aim to determine whether an eight-week supervised, centre-based PR-min programme is non-inferior to a standard eight-week supervised, centre-based PR-gym programme in terms of health outcomes for patients with chronic lung disease.

Methods and analysis: Parallel, two-group, assessor- and statistician-blinded, non-inferiority randomised trial. Participants will be randomised using minimization at the individual level with a 1:1 allocation to PR-min (intervention) or PR-gym (control). Assessment will take place pre-PR (visit 1), post-PR (visit 2) and 12 months following visit 1 (visit 3). Exercise capacity (incremental shuttle walk test), dyspnoea (Chronic Respiratory Questionnaire (CRQ)–Dyspnoea), health-related quality of life (CRQ), frailty (Short Physical Performance Battery), muscle strength (isometric quadriceps maximum voluntary contraction), patient satisfaction (Global Rating of Change Questionnaire), health economic as well as safety and trial process data will be measured. The primary outcome is change in exercise capacity between visit 1 and visit 2. The main statistical analysis will estimate the difference in mean primary and secondary outcomes between patients randomised to PR-gym and PR-min by intention to treat analysis.

Ethics and dissemination: London-Camden and Kings Cross Research Ethics Committee and Health Research Authority have approved the study (18/LO/0315). Results will be submitted for publication in peer-reviewed journals, presented at international conferences, disseminated through social media, patient and public routes and directly shared with stakeholders.

Trial registration: ISRCTN16196765

For peer review only - http://bmjopen.bmj.com/site/about/guidelines.xhtml

Article summary:

Strengths and limitations of this study:

- This is a parallel, two-group, assessor- and statistician-blinded, non-inferiority randomised trial to assess the primary and secondary outcome measures.
- The pulmonary rehabilitation intervention is delivered according to national guidelines: British Thoracic Society Guideline on Pulmonary Rehabilitation in Adults.
- The study includes a 12-month follow-up period to evaluate the long-term effect of the intervention.
- The study includes a health-economic analysis which aims to understand relative cost-effectiveness which may have implications for the NHS and Clinical Commissioning Groups.
- This is a single-centre study based in northwest London and findings may not be applicable to other populations in different locations.

For peer review only

For peer review only - http://bmjopen.bmj.com/site/about/guidelines.xhtml

Introduction:

Background and rationale:

Pulmonary rehabilitation (PR), an evidence-based exercise and education programme, is widely accepted as a cornerstone of management for people with chronic obstructive pulmonary disease (COPD) and other chronic respiratory disorders.[1] The most recent Cochrane review, comprising 65 randomised controlled trials (n=3822), stated that no further trials comparing PR and standard care in patient with COPD were necessary as the evidence supporting the benefits of PR on exercise capacity and health-related quality of life were conclusive.[2]

The majority of evidence to support PR has come from trials conducted in hospital or rehabilitation centres that utilise specialist exercise equipment such as treadmills, cycle ergometers and weights machines (PR-gym).[2] However, in clinical practice, supply does not meet demand, and routine access to specialist equipment may not be feasible.[3] For example, the 2015 Royal College of Physicians National Audit identified that PR services in England and Wales received 68,000 referrals in 2014 out of an estimated 446,000 eligible patients with COPD.[3] Furthermore, 82% of patients enrolled in a PR programme in England and Wales in 2017 were hosted in a community site, of which 61% most likely did not exercise using specialist equipment.[4] Accordingly, exercise training at these sites was completed with minimal equipment (PR-min), typically using portable equipment such as free weights, elastic resistance bands (eg.Theraband™), walking courses, and bodyweight resistance exercises.[3]

Apart from improving accessibility, it has been argued that PR-min may have other advantages over PR-gym. Exercise-training using minimal equipment may better reflect activities of daily-living than training using specialist equipment, and therefore be easier to replicate and maintain at home following discharge from PR. However, it may be more difficult to prescribe and progress exercise, particularly resistance exercise, without the use of specialist equipment.

For peer review only - http://bmjopen.bmj.com/site/about/guidelines.xhtml

A systematic review, conducted by Alison and colleagues, identified eight randomised controlled trials that compared outcomes following exercise interventions completed with minimal equipment to usual care without an exercise intervention in patients with COPD.[5] . The results were conflicting for exercise outcomes. In four studies (n=182) where the six minute walk test (6MWT) was used as the primary outcome measure of exercise capacity, the pooled effect showed a mean (95% confidence interval (CI)) between-group difference of 40 (13 to 67) metres favouring the intervention group.[5] Conversely, in the other four studies (n=389) that used the incremental shuttle walk test (ISW), there was no significant mean (95% CI) between-group difference: 21 (-9 to 51) metres.[5] For health-related quality of life, results were similarly conflicting.[5] Whereas in the four studies that used the St. George's Respiratory Questionnaire there was a sizeable difference between the intervention and control groups (mean (95% CI): -7 (-12 to -3)), the mean between-group difference of the Chronic Respiratory Questionnaire dyspnoea (CRQ-D) and fatigue domains (CRQ-F) did not reach the minimum clinically important difference (MCID) in three other studies.[5] Only one of the eight studies measured muscle strength but results were reported descriptively with no statistical testing.[5] Furthermore, only one of the eight studies would fulfil the BTS guidelines definition of PR,[1] with seven studies not offering any education component.[5]

To our knowledge, no trial has compared supervised centre-based PR using minimal and specialist equipment. A previous National Institute for Health Research Health Technology Assessment-funded randomised 2x2 trial compared PR undertaken in community venues with PR undertaken in a hospital venue, in 240 patients with COPD.[6] Participants were block randomized (hospital n=129; community n=111). Both groups received twice-weekly PR for six weeks with the exercise training protocol identical in both venues. Importantly, neither community nor hospital sites had access to specialist exercise equipment. Patients in both groups improved their walking distance, exceeding the MCID of the endurance shuttle walk test, and there were similar improvements in health-related quality of life.[6] Although the results support the conclusion that the efficacy of PR-min is not influenced by hospital or community locations, this study did not address whether PR-min is non-inferior to 'gold standard' PR-gym. A recent

For peer review only - http://bmjopen.bmj.com/site/about/guidelines.xhtml

trial from Australia compared home-based, lightly supervised PR with minimal access to exercise equipment with gold standard centre-based PR, and demonstrated equivalent outcomes.[7] However there was strong evidence of selection bias in that 42% of eligible participants were not randomised because of a preference for centre-based PR. Furthermore, the changes in the primary outcome (6MWT) following PR in both groups were considerably lower than has been observed in previous meta-analyses,[2] or in national audit data.[4 8] Our group conducted an observational cohort study to compare if an eight-week supervised, centre-based PR-min programme was non-inferior to PR-gym in terms of core PR outcomes in patients with COPD.[9] Using propensity score matching, 318 consecutive patients undergoing PR-min were compared 1:1 with a control group of 318 patients who underwent PR-gym. Similar short-term improvements in exercise capacity and health-related quality of life were observed in both groups (mean (95% CI) difference: ISW: 3 (-16 to 9) metres; CRQ-total: 0.9 (-2.7 to 4.5)). Furthermore, the 95% confidence interval between-group differences for these outcomes did not cross the pre-defined non-inferiority margins. These data suggest that PR-min is non-inferior to PR-gym with regards to short term improvements in exercise capacity and health-related quality of life. However this study was not randomised, and only included short term outcomes. A randomised controlled trial with more varied outcome measures (such as lower limb muscle strength) and longer follow-up is required to confirm these findings.

The aims of the research are to determine whether an eight-week outpatient supervised PR-min programme is non-inferior to a standard eight-week outpatient supervised PR-gym programme in terms of health benefits for patients with chronic respiratory disease.

The primary objective is to determine whether PR-min is non-inferior to PR-gym regarding change in exercise capacity measured using ISW[10] distance from baseline (Visit 1) to post-PR assessment at eight weeks (Visit 2).

The secondary objectives are to: 1) determine whether PR-min is non-inferior to PR-gym regarding changes breathlessness (CRQ–D), health-related quality of life (CRQ) and quadriceps strength (isometric quadriceps

For peer review only - http://bmjopen.bmj.com/site/about/guidelines.xhtml

maximum voluntary contraction) from baseline (Visit 1) to post-PR assessment at eight weeks (Visit 2) and from baseline (Visit 1) to 12 months after the baseline assessment (Visit 3); 2) evaluate the trial process by recording the participant recruitment and retention, participant uptake of PR, PR attendance, PR completion, reasons for PR non-completion and participant satisfaction in each arm of the study, at the appropriate stage of the trial; 3) estimate the cost and cost-effectiveness during the trial period.

Methods and analysis:

<u>Ethical approval</u>

This study has been approved by the Health Research Authority and London Camden and Kings Cross Research Ethics Committee (reference 18/LO/0315). All participants will provide written informed consent.

<u>Study design</u>

This is a parallel, two-group, assessor- and statistician-blinded, non-inferiority, randomised trial. Participants will be randomised at the individual level with a 1:1 allocation to either PR-min or PR-gym. Both interventions will comprise two supervised sessions per week for eight weeks delivered by the same team. Outcome measures will be recorded at baseline assessment for PR (Visit 1), following PR at eight weeks (Visit 2) and at 12 months (Visit 3). The study schema is outlined in figure 1.

<u>Study population</u>

Potential participants will be people referred to Harefield Pulmonary Rehabilitation Unit, Royal Brompton and Harefield NHS Foundation Trust, UK who meet the following inclusion criteria 1) adults >18 years of age; 2) physician diagnosis of stable chronic respiratory disease, typically COPD, interstitial lung disease, bronchiectasis, chronic asthma or chest wall disease; 3) referred for PR in line with BTS guidelines (i.e. ambulatory – can walk ≥5 metres, functional impairment related to breathlessness, typically Medical

For peer review only - http://bmjopen.bmj.com/site/about/guidelines.xhtml

Research Council dyspnoea score ≥2); 4) able to communicate verbally and respond to questions in written English. Potential participants will be excluded if they have 1) any contraindication to moderate intensity physical exercise e.g. unstable cardiovascular disease; 2) progressive cancer or neurological disorder with an expected life expectancy less than 12 months; 3) completed PR within previous 12 months and/ or 4) are unable to provide informed consent.

Consent

Informed consent will be obtained by the nominated researchers as recorded in the Sponsor's Delegation of Responsibilities Log. All individuals taking informed consent will have received consent training. All participants will provide written consent. Consent to enter this study will be obtained after a full account has been provided of its nature, purpose, risks, burdens and potential benefits, and the patient has had the opportunity to deliberate. The patient will be allowed to specify the time they wish to spend deliberating, usually up to 24 hours. The Investigator or designee will explain that the patients are under no obligation to enter the study and that they can withdraw at any time during the study, without having to give a reason. At each visit, the participant's willingness to continue in the study will be ascertained and documented in the research notes. A copy of the consent form is in the online supplement.

Screening and recruitment

Following clinical referral, but prior to pre-PR assessment, potential participants will be screened by a member of the PR team for eligibility to the study. The potential participant will be approached by telephone and provided with a participant information sheet. A member of the research team will obtain informed consent following the pre-PR assessment.

Randomisation, allocation and blinding procedures

Consenting participants will be randomised at the individual level with a 1:1 allocation, using an independent web-based system provided by the United Kingdom Clinical Research Collaboration registered King's Clinical Trials Unit (KCTU), to receive either "usual care" (PR-gym) or intervention (PR-min).

For peer review only - http://bmjopen.bmj.com/site/about/guidelines.xhtml

Randomisation by minimisation will ensure that participant characteristics are balanced between the groups with respect to previous completion of PR (yes/no), multiple deprivation index (most deprived quintile of index: yes/no[11]) and frailty status (Short Physical Performance Battery score < 10/≥10[12]). A proportion of patients will be entered initially using simple randomisation in order to create a level of initial imbalance and the minimisation algorithm will maintain a level of randomness in order to preserve pre-randomisation allocation concealment. Once randomised, the system will automatically generate a full audit trail of the process and send emails to relevant investigators in a blinded or unblinded format, depending on their role. For each arm, participants will be provided with a choice of three sites in northwest London to undertake PR.

Owing to the nature of the interventions, participants and providers of the intervention will not be blinded. However post-rehabilitation assessments will be performed by a researcher blinded to group allocation and not involved in the delivery of either intervention arm.. The trial statistician will also be blinded to group allocation.

<u>Study intervention</u>

PR-gym (control group): The control intervention will be current gold-standard clinical practice. PR-gym will comprise an eight-week outpatient exercise and multi-disciplinary self-management education programme, with two supervised and at least one additional home session each week, and delivered according to the BTS Quality Standards for PR.[13] Supervising staff will comprise specialist respiratory therapists with a minimum of two years' experience in PR. Available equipment will include treadmills, cycle ergometers, cross-trainers, specialist lower limb resistance equipment (e.g. leg press, knee extension). Each supervised session will consist of one hour of exercise (at least 30 minutes aerobic exercise) and 45 minutes of education.

Initial walking speed prescription on the treadmill will be 80% of predicted peak oxygen consumption based on baseline ISW performance[10], whilst initial endurance cycling will be initially set at 60% peak

For peer review only - http://bmjopen.bmj.com/site/about/guidelines.xhtml

workload on a cycle ergometer with the aim of patients completing ten minutes of continuous training. Lower limb resistance training will comprise two sets of ten leg press repetitions on specialist resistance equipment performed with an initial training load of 60% one-repetition maximum. Similarly two sets of ten bilateral knee extension repetitions will be performed on specialist resistance equipment at an initial training load of 60% one-repetition maximum. This will be supplemented with sit-to-stand sets, plus knee lifts/extension and hip abduction with appropriate ankle weights up to 10kg. Upper limb resistance training will comprise biceps curls, shoulder press and upright row with free weights or Therabands™ (red to black). Exercise training will be individualised and regularly progressed (duration and/or intensity) according to standard operating procedures, with targets reviewed at each session. Education will be delivered by a multi-disciplinary team with topics chosen to develop patients' understanding and holistic management of their disease.

PR-min (Intervention group): PR-min will also comprise an eight-week outpatient exercise and multidisciplinary self-management education programme, with two supervised and at least one additional home session each week, and delivered according to the BTS Quality Standards for PR.[13] As for PR-gym, supervising staff will comprise specialist respiratory therapists with at least two years' experience of in PR. There will be no access to treadmills, cycle ergometers, or specialist resistance equipment. Available exercise equipment will include walking circuit, portable steppers, portable pedals, hand and ankle weights (up to 5kg), and Therabands™ (red to black). Each supervised session will consist of one hour of exercise (at least 30 minutes aerobic exercise) and 45 minutes of education.

Initial walking speed prescription will be 80% of predicted peak oxygen consumption based on baseline ISW performance. Participants will be provided with a stopwatch and given time targets to complete a walking course of known distance. Although the resistance of the portable steppers and pedals can be manually adjusted, this cannot be objectively quantified. Initial prescription will be set at "level 1" but individually adjusted to find an intensity where patients can complete ten minutes of continuous training with a target modified Borg breathless score of 3-4 and a Borg rating of Perceived Exertion of 13-15 (on a

For peer review only - http://bmjopen.bmj.com/site/about/guidelines.xhtml
For peer review only

scale of 6-20). Resistance training will include functional activities such as sit-to-stand and step-ups as well as Theraband™ based exercises such as sitting knee extension, leg press and hip flexion as well as standing hip extension, squats, chest press and lateral raise. Progression will be through the use of hand/ankle weights, and increasing resistant Therabands™ (from red to black). Exercise training will be individualised and regularly progressed (either in duration or intensity) with targets reviewed at each supervised session. Education will be delivered by a multi-disciplinary team as per PR-gym.

<u>Study outcome measures</u>

A list of the scheduled outcomes is outlined in table 1 and described in detail in the online supplement.

Table 1. Study outcome measures

	Visit 1	Visit 2	Visit 3
Spirometry	X		X
Frailty (Short Physical Performance Battery)	X	X	X
Exercise capacity (Incremental Shuttle Walk Test)	X	X	X
Dyspnoea (Chronic Respiratory Questionnaire – Dyspnoea domain)	X	X	X
Health-related quality of life (Chronic Respiratory Questionnaire)	X	X	X
Muscle strength (Isometric Quadriceps Maximum Voluntary Contraction)	X	X	X
Patient satisfaction (Global Rating of Change Questionnaire)		X	
Health-economic evaluation (Modified Client Service Receipt Inventory Scale, Euro-Qol-5 Dimensions-5 Levels, NHS Digital - after visit 3 only)	X	X	X
Safety and trial process evaluation	X	X	X

For peer review only - http://bmjopen.bmj.com/site/about/guidelines.xhtml

Sample size calculation

Previous audits of Harefield PR Unit have shown that in participants undergoing PR-gym achieve a mean (standard deviation) change in ISW of 58 (67) metres. The null hypothesis is that PR-min is inferior to the standard treatment PR-gym. The alternative hypothesis is that PR-min is not inferior to PR-gym. The non-inferiority margin will be defined as half the known MCID using the fixed-margin method with a preserved effect of 50% as recommended by previous guidance, including from the United States Food and Drug Administration.[14 15] The MCID of the ISW is 47.5 metres,[16] and therefore 24 metres will be considered the non-inferiority margin. If there is truly no difference between PR-min and PR-gym, then a minimum of 246 patients (123 in each group) is required to be 80% sure that the lower limit of a one-sided 97.5% CI (or equivalently a 95% two-sided CI) will be above the non-inferiority limit of -24 metres. Based on audit data, we anticipate 32% drop out from PR (12% from assessment to starting PR, and 20% from starting PR to completing PR). Taking into account drop-out, the minimum sample size required for analysis will be 362 patients (181 patients per group).

Statistical analysis

For baseline data, continuous and categorical variables will be summarized with descriptive statistics. No significance testing will be carried out. The outcome measures will be described by trial arm and at visits 1, 2 and 3 using descriptive statistics.

The main statistical analyses will estimate the difference in mean primary and secondary outcomes between patients randomised to PR-gym and PR-min by intention to treat principle. Group difference estimates and associated one-sided 97.5% confidence intervals will be reported. The group differences will be compared using one-sided two independent sample t-test or non-parametric equivalent. The significance level is set at one-sided significance level of 0.025.

Regarding missing data the number of baseline variables with complete data will be reported and the primary and secondary outcomes will be analysed as per the recommendations of White et al.[17] Missing

For peer review only - http://bmjopen.bmj.com/site/about/guidelines.xhtml

post-randomisation assessments will be dealt with using an appropriate method of imputation, depending on the missing mechanism.

Adherence is defined as the number of supervised sessions the participant attends irrespective of the assigned intervention (maximum 16). This will be recorded in both intervention arms. As a dichotomous indicator with eight as the cut off. The following variables will be recorded in a 3x2 table according to allocated intervention: 1) compliers always receive the allocated treatment; 2) complete defiers do the opposite of the allocated treatment i.e. attend no sessions in allocated treatment; 3) partial defiers are all others not falling in the above groups. Dropout cause will be recorded using the MORECARE classification of reason for attrition.[18]

In addition to the primary intention-to-treat analysis, the effect of actually receiving treatment as defined in the protocol will also be estimated. If non-compliance with is >10% a Complier-Average Causal-Effect (CACE) will be estimated.[19] The following sensitivity analyses are planned: 1) per protocol; 2) including only those in the upper quartile for baseline ISW; 3) complete case; 4) complier Average Causal Effects (CACE) analysis[19] and 5) primary diagnosis of COPD.

Data collection, management and monitoring

A web based electronic data capture (EDC) system has been designed using the InferMed Macro 4 system and will be maintained by KCTU for the duration of the project. Source data will be entered by recruiting site staff, typically within one week of data collection by authorised staff onto the EDC. A full audit trial of data entry and any subsequent changes to entered data will be automatically date and time stamped, alongside information about the user making the entry/changes within the system.

The primary investigator (WM) will take overall responsibility for the conduct of MISTER. The trial co-ordinator (CMN) will supervise the day-to-day operation of the project and is responsible for ensuring that Good Clinical Practice guidelines are followed. An unblinded member of the MISTER research team (JAW) will monitor the data and review a random sample of 10% of completed case report forms against clinical

For peer review only - http://bmjopen.bmj.com/site/about/guidelines.xhtml

records. Monitoring will ensure protocol compliance, proper study management and timely completion of study procedures.

Monitoring and auditing

A Data Monitoring and Ethics Committee (DMEC) has been appointed. The members of this committee are not members of the applicants' or sponsors' institution and include an academic Consultant Respiratory Physician and an independent statistician.

The requirement for study monitoring or audit will be based on the Sponsor's Research Office risk assessment procedure and applicable standard operating procedures. It is the responsibility of the Research Office to determine the monitoring risk assessment and explain the rationale to the study research team.

Patient and public involvement (PPI)

The concept of the research project arose directly from PPI, and the development of this project has included PPI throughout each stage. We have a named PPI co-applicant who will continue to be closely involved in the design and management of the research through membership of the Trial Steering Group that will meet at the beginning of the research period, and then every six months throughout the data collection/analysis and dissemination period. This PPI member will be joined by a second representative and will meet the project manager at regular intervals throughout the study. The PPI group will provide input into written material for patients, how results data are presented, particularly to lay audiences and will have a role in dissemination of research findings.

Safety monitoring

A Serious Adverse Event (SAE) is defined as an untoward occurrence that a) results in death; b) is life-threatening; c) requires hospitalization or prolongation of existing hospitalization; d) results in persistent or significant disability or incapacity or e) is otherwise considered medically significant by the investigator.

For peer review only - http://bmjopen.bmj.com/site/about/guidelines.xhtml

The Investigator and research team are responsible for reporting events to the Research Office immediately and/or within 24 hours of becoming aware of the event. An SAE occurring to a research participant will be reported to the Research Ethics Committee that gave a favorable opinion of the study.

Amendment

Should the authors wish to make any substantial amendments to the application or supporting documentation, a notice of amendment will be submitted to the Health Research Authority for consideration.

Dissemination:

We will utilise a broad strategy to maximise dissemination of our findings: 1) sharing of scientific findings via open-access publication in high impact journals and presentation at international meetings; 2) plain English summaries of findings for public bodies and web-sites (e.g. NIHR, CLARHC, British Lung Foundation, patient.co.uk) to communicate evidence through a user-friendly interface; 3) online pages about the project on websites of contributing organisations (Royal Brompton & Harefield NHS Foundation Trust, Cicely Saunders Institute, King's College London); 4) social media (e.g. Twitter); 5) public engagement via talks with service user groups and open public events so patients and their caregivers can learn about the research; 6) direct sharing of findings with public bodies (e.g. National Horizon Scanning Research and Intelligence Centre) and policy makers to whom the applicants have direct access (e.g. BTS Pulmonary Rehabilitation Advisory Group, European Respiratory Society (ERS), American Thoracic Society (ATS), Society for Research in Rehabilitation) to facilitate uptake of findings into research strategy and policy; 7) education of commissioners to influence future NHS service delivery.

For peer review only - http://bmjopen.bmj.com/site/about/guidelines.xhtml

Discussion:

PR is a core strategy in the management for COPD and other chronic respiratory disorders.[1] The majority of evidence to support PR has come from trials conducted in locations that utilise specialist exercise equipment.[2] Data from a national audit indicate that many PR programmes in England and Wales do not have access to specialist exercise equipment with exercise training completed using simple equipment.[3] There is a paucity of robust literature examining the efficacy of PR-min and to our knowledge, there have been no trials comparing supervised centre-based PR-min and PR-gym. One observational study demonstrated that PR-min is non-inferior to PR-gym in terms of exercise capacity and health-related quality of life but indicating that further investigation using a randomised controlled trial is.[9] Therefore, this study aims to bridge the gap in knowledge by identifying whether PR-min is non-inferior to PR-gym regarding change in core PR outcome measures. This is an important and current health service question given the increasing disparity between supply and demand of PR that has implications for the delivery and future development of this programme at national and international levels.

The strengths of this study include the design: a parallel, two-group, assessor- and statistician-blinded, non-inferiority randomised trial which is the appropriate design to assess the primary and secondary outcome measures. The intervention, PR, is delivered according to national guidelines and the population of interest is people with chronic respiratory disease, both of which mean the results will be generalisable. In addition, study involves a 12-month follow-up period to evaluate the long-term effect of the intervention and a health-economic analysis which aims to understand the potential cost-effectiveness of the trial which may have implications for the NHS and Clinical Commissioning Groups. A limitation of the study is that it is a single-centre study based in northwest London which may mean that the findings may not be generalisable to other populations in different locations.

For peer review only - http://bmjopen.bmj.com/site/about/guidelines.xhtml

Acknowledgements

We would like to acknowledge Joanna Kelly, Evangelos Georgio, Janice Jimenez and their teams in the Kings Clinical Trials Unit data centre who developed and will maintain the randomisation and Elsevier MACRO database systems for the trial. We would also like to acknowledge Ms Peihan Yu, statistician at Kings College London, for her help developing the study protocol. We would like to express our gratitude to the Harefield Pulmonary Rehabilitation Unit, Royal Brompton and Harefield NHS Foundation Trust who will help with participant identification and delivery of the pulmonary rehabilitation programme.

For peer review only

Figure legend

Figure 1. Study schema.

For peer review only - http://bmjopen.bmj.com/site/about/guidelines.xhtml

Full references

1. Bolton CE, Blakey JD, Morgan MD. The British Thoracic Society guideline on pulmonary rehabilitation in adults: your opinion is noted. *Thorax* 2014;69(4):388-9. doi: 10.1136/thoraxjnl-2013-204754 [published Online First: 2014/01/17]
2. McCarthy B, Casey D, Devane D, et al. Pulmonary rehabilitation for chronic obstructive pulmonary disease. *The Cochrane database of systematic reviews* 2015;2 doi: 10.1002/14651858.CD003793.pub3 [published Online First: 2015/02/24]
3. RCP B. National COPD Audit Programme. Pulmonary Rehabilitation: Steps to Breathe Better 2016 [Available from: file:///C:/Users/cn805/Downloads/Nat%20COPD%20PR%20Clinical%20Audit%202015%20Full%20report_0.pdf accessed 4th December 2017.
4. Steiner M, McMillan V, Lowe D, et al. Pulmonary rehabilitation: an exercise in improvement. *National chronic obstructive pulmonary disease (COPD) audit programme: clinical and organisational audits of pulmonary rehabilitation services in England and Wales* 2017
5. Alison JA, McKeough ZJ. Pulmonary rehabilitation for COPD: are programs with minimal exercise equipment effective? *Journal of thoracic disease* 2014;6(11):1606.
6. Waterhouse J, Walters S, Oluboyede Y, et al. A randomised 2 x 2 trial of community versus hospital pulmonary rehabilitation, followed by telephone or conventional follow-up. *Health technology assessment (Winchester, England)* 2010;14(6):i-v, vii-xi, 1-140.
7. Holland AE, Mahal A, Hill CJ, et al. Home-based rehabilitation for COPD using minimal resources: a randomised, controlled equivalence trial. *Thorax* 2017;72(1):57-65.
8. NACAP. National Asthma and COPD Audit Programme: Pulmonary rehabilitation clinical audit interim report 2020 [Available from: https://www.rcplondon.ac.uk/projects/outputs/national-asthma-and-copd-audit-programme-nacap-pulmonary-rehabilitation-clinical accessed 17th August 2020.
9. Patel S, Palmer MD, Nolan CM, et al. Supervised pulmonary rehabilitation using minimal or specialist exercise equipment in COPD: a propensity-matched analysis. *Thorax* 2020 doi: 10.1136/thoraxjnl-2020-215281 [published Online First: 2020/11/03]
10. Singh SJ, Morgan MD, Scott S, et al. Development of a shuttle walking test of disability in patients with chronic airways obstruction. *Thorax* 1992;47(12):1019-24.
11. Ministry for Housing Communities and Local Government. English Indices of Deprivation 2015 2015 [Available from: http://imd-by-postcode.opendatacommunities.org/imd/2015 accessed 3rd November 2020.
12. European Medicines Agency. Reflection paper on physical frailty: instruments for baseline characterisation of older populations in clinical

trials 2018 [Available from: https://www.ema.europa.eu/en/documents/scientific-guideline/reflection-paper-physical-frailty-instruments-baseline-characterisation-older-populations-clinical_en.pdf accessed 22nd October 2020.
13. BTS. Quality Standards for Pulmonary Rehabilitation. *British Thoracic Society Reports* 2014;6(2)
14. Piaggio G, Elbourne DR, Pocock SJ, et al. Reporting of noninferiority and equivalence randomized trials: extension of the CONSORT 2010 statement. *Jama* 2012;308(24):2594-604.
15. Wangge G, Putzeist M, Knol MJ, et al. Regulatory scientific advice on non-inferiority drug trials. *PloS one* 2013;8(9):e74818.
16. Singh SJ, Jones PW, Evans R, et al. Minimum clinically important improvement for the incremental shuttle walking test. *Thorax* 2008;63(9):775-7.
17. White IR, Horton NJ, Carpenter J, et al. Strategy for intention to treat analysis in randomised trials with missing outcome data. *Bmj* 2011;342:d40.
18. Higginson IJ, Evans CJ, Grande G, et al. Evaluating complex interventions in end of life care: the MORECare statement on good practice generated by a synthesis of transparent expert consultations and systematic reviews. *BMC medicine* 2013;11(1):111.
19. Jo B. Statistical power in randomized intervention studies with noncompliance. *Psychological Methods* 2002;7(2):178.

For peer review only - http://bmjopen.bmj.com/site/about/guidelines.xhtml

Authors' contributions: CMN, MM and WD-CM drafted the manuscript. WG, RW, MM, CMN and WD-CM drafted the statistical analysis plan. All authors reviewed the final draft of the manuscript.

Funding statement: This work is supported by National Institute for Health Research grant number PB-PG-0816-20022.

Competing interests statement

JAW, SP, REB, OP, RW, WG have no competing interests

CMN reports fees from Novarits outside of this research.

MM consultancy fees from Fresenius Kabi and Helsinn, grants from National Institute for Health Research, grants from British Lung Foundation, outside the submitted work.

WD-CM reports personal fees from Jazz Pharmaceuticals, personal fees from Mundipharma, personal fees from Novartis, grants from Pfizer, non-financial support from GSK, grants from National Institute for Health Research, grants from British Lung Foundation, outside the submitted work.

Word Count: 3879

For peer review only - http://bmjopen.bmj.com/site/about/guidelines.xhtml

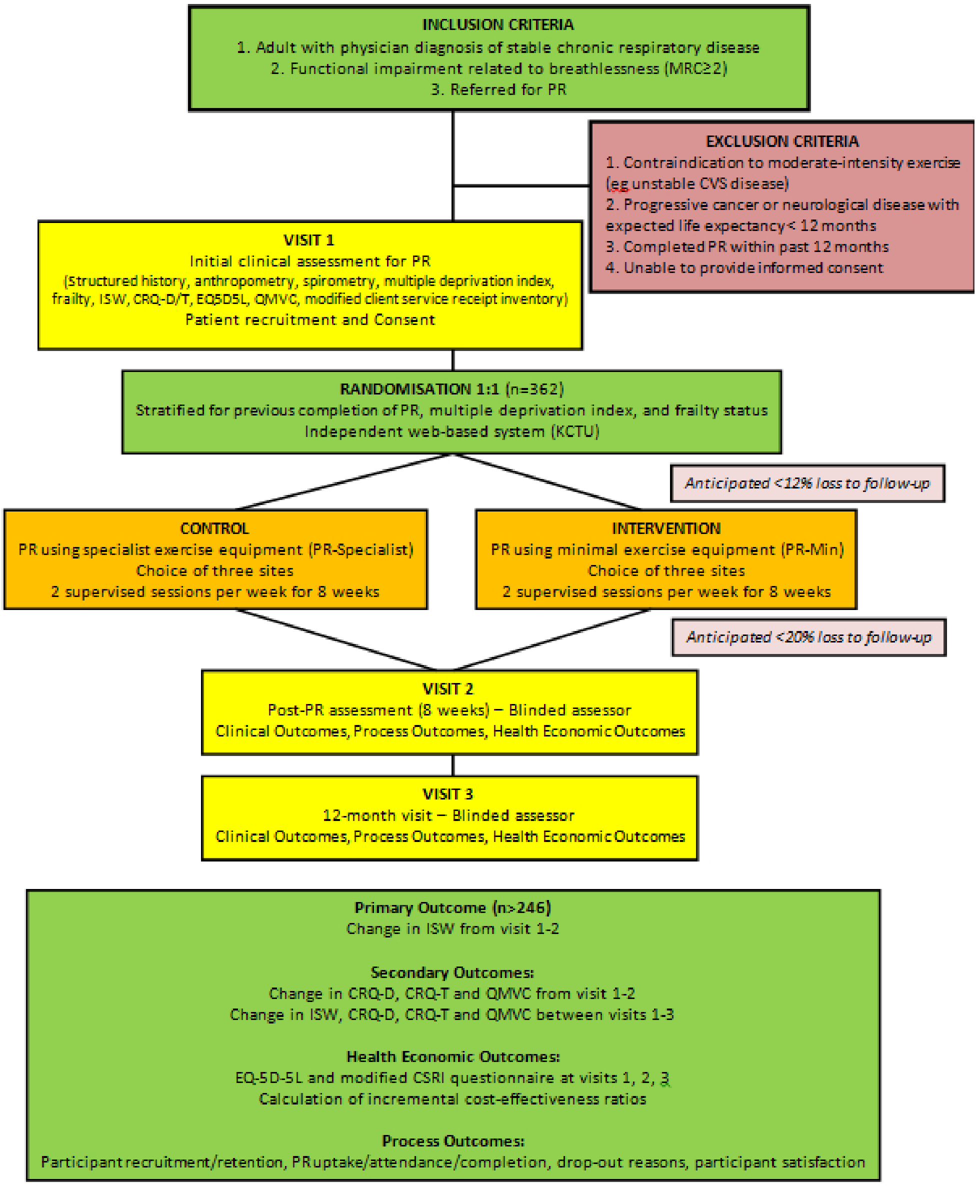

Figure 1. Study schema.

MInimal versus SpecialisT Equipment in the delivery of pulmonary Rehabilitation (MISTER): a randomised controlled trial

Online supplement

Consent: Informed consent form

Royal Brompton & Harefield NHS
NHS Foundation Trust

PARTICIPANT CONSENT FORM

MInimal versus SpecialisT Equipment in the delivery of pulmonary Rehabilitation (MISTER): a randomised controlled trial

IRAS Reference: 241564

Name of Researcher: ....

Participant study ID number: ....

Please initial the boxes

1. I confirm that I have read and understand the participant information leaflet (version **3** dated **01/10/2018**) for the above study. I have had the opportunity to consider the information, ask questions and have had these answered satisfactorily. ☐

2. I understand that my participation is voluntary and that I am free to withdraw at any time without giving any reason, although data collected until that point may be used for research purposes with my agreement, without my medical care or legal rights being affected. ☐

3. I understand that sections of my medical notes relating to the study may be looked at by regulatory bodies conducting inspections which may include members of staff within the NHS Trust who are outside of the immediate clinical and research teams. I give permission for these individuals to have access to my records. ☐

4. a) I agree that the information held and maintained by NHS Digital and other NHS bodies will be used to provide information about my healthcare use, and in the case of my death, information about this. ☐

   b) I consent to share identifiable details (name, NHS number, date of birth, post-code) with NHS Digital. ☐

5. I consent to allowing my postcode to be used to calculate the Multiple Deprivation Index. ☐

6. I agree to my GP being informed of my participation in the study. ☐

7. I agree to take part in the above study. ☐

8. I wish to receive feedback on the study results when available. ☐

Name of Patient	Date	Signature
Name of Person taking consent (If different from researcher)	Date	Signature
Researcher	Date	Signature

When completed: 1 for patient; 1 (original) for researcher site file; 1 to be kept in medical notes

Study outcome measures

Spirometry: Visit 1 and visit 3

All participants will undergo spirometry performing the Forced Vital Capacity (FVC) / Forced Expiratory Volume in one second (FEV_1) manoeuvre as described in Appendix 1, using an EasyOne™ diagnostic spirometer. As a minimum, three technically acceptable tests must be performed and must meet the Association of Respiratory Technology and Physiology reproducibility criteria.[11]. Results obtained will include FVC, FEV_1 and the FEV_1/FVC ratio. Percent predicted reference equations from the European Respiratory Society/European Steel and Coal Society were used.[12]

Frailty: Visit 1, visit 2 and visit 3

All participants will perform the Short Physical Performance Battery (SPPB) which is a simple test of lower limb functional performance and a marker of frailty.[13] It comprises an assessment of standing balance, usual walking speed and ability to stand from a chair. The SPPB is scored out of 12 with a higher score indicating better functional performance. A score of <10 indicates a frail status and this cut-off will be used in the randomisation procedure.

Exercise capacity: Visit 1, visit 2 and visit 3

The ISW is an incremental, externally paced, field walking test that involves participants walking around a 10 metre course in time to a series of progressively faster beeps played from a CD player. At visit 1**,** two tests (a practice test and then the formal test) will be undertaken with at least 30 minutes rest between tests.[14] This is in accordance with international technical standards. Measures of breathlessness, oxygen saturation levels and heart rate will be recorded before and after the test. The distance completed in metres will also be recorded.

Dyspnoea: Visit 1, visit 2 and visit 3

Dyspnoea will be measured using the CRQ-D.[15] The dyspnoea domain allows the patients to choose five activities that have been limited by shortness of breath in the past two weeks.

Health-related quality of life: Visit 1, visit 2 and visit 3

Health-related quality of life will be measured using the CRQ.[15] This 20 item questionnaire, is responsive to PR, and contains four domains; dyspnea (described above), fatigue, emotional function and mastery, and a total score. Each item is scored on a seven point Likert scale, with a lower score indicating a higher symptom burden.

Muscle strength: Visit 1, visit 2 and visit 3

Isometric quadriceps maximal voluntary contraction of the dominant leg will be measured using specially designed chair and strain gauge. This test involves the participant pushing against an ankle strap with the knee positioned at 90^0.[16] A warm-up and six efforts will be performed.

For peer review only

Patient satisfaction: Visit 2

To measure patient satisfaction, participants will rate their response to the following question *"How do you feel your overall condition has changed after rehabilitation?"* on a five point Global Rating of Change Questionnaire. The scale ranges from *"1: I feel much better"* to *"5: I feel much worse".*

Health economic evaluation: Visit 1, visit 2 and visit 3

- The Modified Client Service Receipt Inventory questionnaire will be used to record information on health care resource, medicine and equipment use, informal care provided by family members, time off work and costs borne by the patient and family relating to the chronic lung disease.
- The EQ5D5L is a generic measure of health status that comprises a visual analogue scale and five-item questionnaire with the following domains: mobility; self-care; usual activities; pain/discomfort and anxiety/depression.[17]
- After visit 3, data will be obtained from NHS Digital regarding health resource utilization between visit 1 and visit 3. This will include information on elective and non-elective hospital admissions (e.g. reason for admission; type of ward; treatment received; length of stay (including date of admission and discharge); location of discharge) as well as outpatient healthcare contacts (e.g. type of appointment, number of contacts, treatment received) and mortality (e.g. date of death, reason for death, place of death).

Safety and trial process evaluation: Visit 1 and visit 2

- Safety will be assessed in real-time using adverse event reporting.
- PR uptake, adherence and completion will be assessed objectively through PR attendance registers and training records. Reasons for non-completion of PR will be assessed objectively through PR records.

For peer review only

SPIRIT

STANDARD PROTOCOL ITEMS: RECOMMENDATIONS FOR INTERVENTIONAL TRIALS

SPIRIT 2013 Checklist: Recommended items to address in a clinical trial protocol and related documents*

Section/item	Item No	Description
**Administrative information**		
Title	1	Descriptive title identifying the study design, population, interventions, and, if applicable, trial acronym
Trial registration	2a	Trial identifier and registry name. If not yet registered, name of intended registry
	2b	All items from the World Health Organization Trial Registration Data Set
Protocol version	3	Date and version identifier
Funding	4	Sources and types of financial, material, and other support
Roles and responsibilities	5a	Names, affiliations, and roles of protocol contributors
	5b	Name and contact information for the trial sponsor
	5c	Role of study sponsor and funders, if any, in study design; collection, management, analysis, and interpretation of data; writing of the report; and the decision to submit the report for publication, including whether they will have ultimate authority over any of these activities
	5d	Composition, roles, and responsibilities of the coordinating centre, steering committee, endpoint adjudication committee, data management team, and other individuals or groups overseeing the trial, if applicable (see Item 21a for data monitoring committee)
**Introduction**		
Background and rationale	6a	Description of research question and justification for undertaking the trial, including summary of relevant studies (published and unpublished) examining benefits and harms for each intervention
	6b	Explanation for choice of comparators
Objectives	7	Specific objectives or hypotheses
Trial design	8	Description of trial design including type of trial (eg, parallel group, crossover, factorial, single group), allocation ratio, and framework (eg, superiority, equivalence, noninferiority, exploratory)

For peer review only

Methods: Participants, interventions, and outcomes

Study setting	9	Description of study settings (eg, community clinic, academic hospital) and list of countries where data will be collected. Reference to where list of study sites can be obtained	
Eligibility criteria	10	Inclusion and exclusion criteria for participants. If applicable, eligibility criteria for study centres and individuals who will perform the interventions (eg, surgeons, psychotherapists)	
Interventions	11a	Interventions for each group with sufficient detail to allow replication, including how and when they will be administered	
	11b	Criteria for discontinuing or modifying allocated interventions for a given trial participant (eg, drug dose change in response to harms, participant request, or improving/worsening disease)	
	11c	Strategies to improve adherence to intervention protocols, and any procedures for monitoring adherence (eg, drug tablet return, laboratory tests)	
	11d	Relevant concomitant care and interventions that are permitted or prohibited during the trial	
Outcomes	12	Primary, secondary, and other outcomes, including the specific measurement variable (eg, systolic blood pressure), analysis metric (eg, change from baseline, final value, time to event), method of aggregation (eg, median, proportion), and time point for each outcome. Explanation of the clinical relevance of chosen efficacy and harm outcomes is strongly recommended	
Participant timeline	13	Time schedule of enrolment, interventions (including any run-ins and washouts), assessments, and visits for participants. A schematic diagram is highly recommended (see Figure)	
Sample size	14	Estimated number of participants needed to achieve study objectives and how it was determined, including clinical and statistical assumptions supporting any sample size calculations	
Recruitment	15	Strategies for achieving adequate participant enrolment to reach target sample size	

Methods: Assignment of interventions (for controlled trials)

Allocation:

Sequence generation	16a	Method of generating the allocation sequence (eg, computer-generated random numbers), and list of any factors for stratification. To reduce predictability of a random sequence, details of any planned restriction (eg, blocking) should be provided in a separate document that is unavailable to those who enrol participants or assign interventions	

Allocation concealment mechanism	16b	Mechanism of implementing the allocation sequence (eg, central telephone; sequentially numbered, opaque, sealed envelopes), describing any steps to conceal the sequence until interventions are assigned
Implementation	16c	Who will generate the allocation sequence, who will enrol participants, and who will assign participants to interventions
Blinding (masking)	17a	Who will be blinded after assignment to interventions (eg, trial participants, care providers, outcome assessors, data analysts), and how
	17b	If blinded, circumstances under which unblinding is permissible, and procedure for revealing a participant's allocated intervention during the trial
**Methods: Data collection, management, and analysis**		
Data collection methods	18a	Plans for assessment and collection of outcome, baseline, and other trial data, including any related processes to promote data quality (eg, duplicate measurements, training of assessors) and a description of study instruments (eg, questionnaires, laboratory tests) along with their reliability and validity, if known. Reference to where data collection forms can be found, if not in the protocol
	18b	Plans to promote participant retention and complete follow-up, including list of any outcome data to be collected for participants who discontinue or deviate from intervention protocols
Data management	19	Plans for data entry, coding, security, and storage, including any related processes to promote data quality (eg, double data entry; range checks for data values). Reference to where details of data management procedures can be found, if not in the protocol
Statistical methods	20a	Statistical methods for analysing primary and secondary outcomes. Reference to where other details of the statistical analysis plan can be found, if not in the protocol
	20b	Methods for any additional analyses (eg, subgroup and adjusted analyses)
	20c	Definition of analysis population relating to protocol non-adherence (eg, as randomised analysis), and any statistical methods to handle missing data (eg, multiple imputation)
**Methods: Monitoring**		
Data monitoring	21a	Composition of data monitoring committee (DMC); summary of its role and reporting structure; statement of whether it is independent from the sponsor and competing interests; and reference to where further details about its charter can be found, if not in the protocol. Alternatively, an explanation of why a DMC is not needed

For peer review only - http://bmjopen.bmj.com/site/about/guidelines.xhtml

	21b	Description of any interim analyses and stopping guidelines, including who will have access to these interim results and make the final decision to terminate the trial
Harms	22	Plans for collecting, assessing, reporting, and managing solicited and spontaneously reported adverse events and other unintended effects of trial interventions or trial conduct
Auditing	23	Frequency and procedures for auditing trial conduct, if any, and whether the process will be independent from investigators and the sponsor
**Ethics and dissemination**		
Research ethics approval	24	Plans for seeking research ethics committee/institutional review board (REC/IRB) approval
Protocol amendments	25	Plans for communicating important protocol modifications (eg, changes to eligibility criteria, outcomes, analyses) to relevant parties (eg, investigators, REC/IRBs, trial participants, trial registries, journals, regulators)
Consent or assent	26a	Who will obtain informed consent or assent from potential trial participants or authorised surrogates, and how (see Item 32)
	26b	Additional consent provisions for collection and use of participant data and biological specimens in ancillary studies, if applicable
Confidentiality	27	How personal information about potential and enrolled participants will be collected, shared, and maintained in order to protect confidentiality before, during, and after the trial
Declaration of interests	28	Financial and other competing interests for principal investigators for the overall trial and each study site
Access to data	29	Statement of who will have access to the final trial dataset, and disclosure of contractual agreements that limit such access for investigators
Ancillary and post-trial care	30	Provisions, if any, for ancillary and post-trial care, and for compensation to those who suffer harm from trial participation
Dissemination policy	31a	Plans for investigators and sponsor to communicate trial results to participants, healthcare professionals, the public, and other relevant groups (eg, via publication, reporting in results databases, or other data sharing arrangements), including any publication restrictions
	31b	Authorship eligibility guidelines and any intended use of professional writers
	31c	Plans, if any, for granting public access to the full protocol, participant-level dataset, and statistical code

For peer review only

Appendices

Informed consent materials	32	Model consent form and other related documentation given to participants and authorised surrogates
Biological specimens	33	Plans for collection, laboratory evaluation, and storage of biological specimens for genetic or molecular analysis in the current trial and for future use in ancillary studies, if applicable

*It is strongly recommended that this checklist be read in conjunction with the SPIRIT 2013 Explanation & Elaboration for important clarification on the items. Amendments to the protocol should be tracked and dated. The SPIRIT checklist is copyrighted by the SPIRIT Group under the Creative Commons "Attribution-NonCommercial-NoDerivs 3.0 Unported" license.

For peer review only

BMJ Open

Minimal versus specialist equipment in the delivery of pulmonary rehabilitation: Protocol for a non-inferiority randomised controlled trial

Journal:	*BMJ Open*
Manuscript ID	bmjopen-2020-047524.R1
Article Type:	Protocol
Date Submitted by the Author:	11-May-2021
Complete List of Authors:	Nolan, Claire; Guy's and St Thomas' Hospitals NHS Trust, Harefield Respiratory Research Group; Imperial College London, National Heart and Lung Institute<br>Walsh, Jessica; Guy's and St Thomas' Hospitals NHS Trust, Harefield Respiratory Research Group<br>Patel, Suhani; Guy's and St Thomas' Hospitals NHS Trust, Harefield Respiratory Research Group; Imperial College London, National Heart and Lung Institute<br>Barker, Ruth; Guy's and St Thomas' Hospitals NHS Trust, Harefield Respiratory Research Group; Imperial College London, National Heart and Lung Institute<br>Polgar, Oliver; Guy's and St Thomas' NHS Foundation Trust, Harefield Respiratory Research Group<br>Maddocks, Matthew; King's College London, Cicely Saunders Institute, Division of Palliative Care, Policy & Rehabilitation<br>Gao, Wei; King's College London, Cicely Saunders Institute, Division of Palliative Care, Policy & Rehabilitation<br>Wilson, Rebecca; King's College London, Cicely Saunders Institute, Division of Palliative Care, Policy & Rehabilitation<br>Man, William; Imperial College London, National Heart and Lung Institute; Guy's and St Thomas' Hospitals NHS Trust, Harefield Respiratory Research Group
**Primary Subject Heading**:	Rehabilitation medicine
Secondary Subject Heading:	Rehabilitation medicine, Respiratory medicine
Keywords:	REHABILITATION MEDICINE, Chronic airways disease < THORACIC MEDICINE, Emphysema < THORACIC MEDICINE, Interstitial lung disease < THORACIC MEDICINE

SCHOLARONE™
Manuscripts

I, the Submitting Author has the right to grant and does grant on behalf of all authors of the Work (as defined in the below author licence), an exclusive licence and/or a non-exclusive licence for contributions from authors who are: i) UK Crown employees; ii) where BMJ has agreed a CC-BY licence shall apply, and/or iii) in accordance with the terms applicable for US Federal Government officers or employees acting as part of their official duties; on a worldwide, perpetual, irrevocable, royalty-free basis to BMJ Publishing Group Ltd ("BMJ") its licensees and where the relevant Journal is co-owned by BMJ to the co-owners of the Journal, to publish the Work in this journal and any other BMJ products and to exploit all rights, as set out in our licence.

The Submitting Author accepts and understands that any supply made under these terms is made by BMJ to the Submitting Author unless you are acting as an employee on behalf of your employer or a postgraduate student of an affiliated institution which is paying any applicable article publishing charge ("APC") for Open Access articles. Where the Submitting Author wishes to make the Work available on an Open Access basis (and intends to pay the relevant APC), the terms of reuse of such Open Access shall be governed by a Creative Commons licence – details of these licences and which Creative Commons licence will apply to this Work are set out in our licence referred to above.

Other than as permitted in any relevant BMJ Author's Self Archiving Policies, I confirm this Work has not been accepted for publication elsewhere, is not being considered for publication elsewhere and does not duplicate material already published. I confirm all authors consent to publication of this Work and authorise the granting of this licence.

For peer review only

Minimal versus specialist equipment in the delivery of pulmonary rehabilitation: Protocol for a non-inferiority randomised controlled trial

Dr Claire M. Nolan PhD [1,2] c.nolan@rbht.nhs.uk

Mrs Jessica A Walsh [1] j.walsh@rbht.nhs.uk

Ms. Suhani Patel [1] s.patel1@rbht.nhs.uk

Ms Ruth E. Barker [1,2] r.barker2@rbht.nhs.uk

Mr Oliver Polgar [1] o.polgar@rbht.nhs.uk

Dr Matthew Maddocks PhD [3] matthew.maddocks@kcl.ac.uk

Dr Wei Gao PhD [3] wei.gao@kcl.ac.uk

Dr Rebecca Wilson PhD [3] rebecca.wilson@kcl.ac.uk

Dr William D-C. Man PhD [1,2,4] w.man@rbht.nhs.uk

Affiliations:

1. Harefield Respiratory Research Group, Royal Brompton and Harefield Clinical Group, Guy's and St Thomas' NHS Foundation Trust, UK
2. National Heart and Lung Institute, Imperial College London, UK
3. King's College London, Cicely Saunders Institute, Division of Palliative Care, Policy & Rehabilitation, London, UK
4. Harefield Pulmonary Rehabilitation Unit, Royal Brompton and Harefield Clinical Group, Guy's and St Thomas' NHS Foundation Trust, UK

For peer review only

Corresponding author: Dr Claire Nolan, Senior Research Physiotherapist, Royal Brompton and Harefield Clinical Group, Guy's and St Thomas' NHS Foundation Trust, Harefield Hospital, Middlesex, UB9 6JH, United Kingdom, Tel: +44 (0)1895 823737, Fax: +44 (0)1895 828889, Email: c.nolan@rbht.nhs.uk

Abstract

Introduction: Pulmonary rehabilitation (PR), an exercise and education programme for people with chronic lung disease, aims to improve exercise capacity, breathlessness and quality of life. Most evidence to support PR is from trials that utilise specialist exercise equipment e.g. treadmills (PR-gym). However, a significant proportion of programmes do not have access to specialist equipment with training completed with minimal exercise equipment (PR-min). There is a paucity of robust literature examining the efficacy of supervised, centre-based PR-min. We aim to determine whether an eight-week supervised, centre-based PR-min programme is non-inferior to a standard eight-week supervised, centre-based PR-gym programme in terms of exercise capacity and health outcomes for patients with chronic lung disease.

Methods and analysis: Parallel, two-group, assessor- and statistician-blinded, non-inferiority randomised trial. 436 participants will be randomised using minimization at the individual level with a 1:1 allocation to PR-min (intervention) or PR-gym (control). Assessment will take place pre-PR (visit 1), post-PR (visit 2) and 12 months following visit 1 (visit 3). Exercise capacity (incremental shuttle walk test), dyspnoea (Chronic Respiratory Questionnaire (CRQ)–Dyspnoea), health-related quality of life (CRQ), frailty (Short Physical Performance Battery), muscle strength (isometric quadriceps maximum voluntary contraction), patient satisfaction (Global Rating of Change Questionnaire), health economic as well as safety and trial process data will be measured. The primary outcome is change in exercise capacity between visit 1 and visit 2. Two sample t-tests on an intention to treat basis will be used to estimate the difference in mean primary and secondary outcomes between patients randomised to PR-gym and PR-min.

Ethics and dissemination: London-Camden and Kings Cross Research Ethics Committee and Health Research Authority have approved the study (18/LO/0315). Results will be submitted for publication in

For peer review only

For peer review only - http://bmjopen.bmj.com/site/about/guidelines.xhtml

peer-reviewed journals, presented at international conferences, disseminated through social media, patient and public routes and directly shared with stakeholders.

Trial registration: ISRCTN16196765

Article summary:

Strengths and limitations of this study:

- This is a parallel, two-group, assessor- and statistician-blinded, non-inferiority randomised trial to assess the primary and secondary outcome measures.
- The pulmonary rehabilitation intervention is delivered according to national guidelines: British Thoracic Society Guideline on Pulmonary Rehabilitation in Adults.
- The study includes a 12-month follow-up period to evaluate the long-term effect of the intervention.
- The study includes a health-economic analysis which aims to understand relative cost-effectiveness which may have implications for NHS commissioning.
- This is a single-centre study based in northwest London and findings may not be applicable to other populations in different locations.

For peer review only - http://bmjopen.bmj.com/site/about/guidelines.xhtml

Introduction:

Background and rationale:

Pulmonary rehabilitation (PR), an evidence-based exercise and education programme, is widely accepted as a cornerstone of management for people with chronic obstructive pulmonary disease (COPD) and other chronic respiratory disorders.[1] The most recent Cochrane review, comprising 65 randomised controlled trials (n=3822), stated that no further trials comparing PR and standard care in patient with COPD were necessary as the evidence supporting the benefits of PR on exercise capacity and health-related quality of life were conclusive.[2]

The majority of evidence to support PR has come from trials conducted in hospital or rehabilitation centres that utilise specialist exercise equipment such as treadmills, cycle ergometers and weights machines (PR-gym).[2] However, in clinical practice, supply does not meet demand, and routine access to specialist equipment may not be feasible.[3] For example, the 2015 Royal College of Physicians National Audit identified that PR services in England and Wales received 68,000 referrals in 2014 out of an estimated 446,000 eligible patients with COPD.[3] Furthermore, 82% of patients enrolled in a PR programme in England and Wales in 2017 were hosted in a community site such as a community centre or church hall, of which 61% most likely did not exercise using specialist equipment.[4] Accordingly, exercise training at these sites, although supervised by PR professionals, was completed with minimal equipment (PR-min), typically using portable equipment such as free weights, elastic resistance bands (eg.Theraband™), walking courses, and bodyweight resistance exercises.[3]

Apart from improving accessibility, it has been argued that PR-min may have other advantages over PR-gym. Exercise-training using minimal equipment may better reflect activities of daily-living than training using specialist equipment, and therefore be easier to replicate and maintain at home following discharge

For peer review only - http://bmjopen.bmj.com/site/about/guidelines.xhtml

from PR. However, it may be more difficult to prescribe and progress exercise, particularly resistance exercise, without the use of specialist equipment.

A systematic review, conducted by Alison and colleagues, identified eight randomised controlled trials that compared outcomes following exercise interventions completed with minimal equipment to usual care without an exercise intervention in patients with COPD.[5] . The results were conflicting for exercise outcomes. In four studies (n=182) where the six minute walk test (6MWT) was used as the primary outcome measure of exercise capacity, the pooled effect showed a mean (95% confidence interval (CI)) between-group difference of 40 (13 to 67) metres favouring the intervention group.[5] Conversely, in the other four studies (n=389) that used the incremental shuttle walk test (ISW), there was no significant mean (95% CI) between-group difference: 21 (-9 to 51) metres.[5] For health-related quality of life, results were similarly conflicting.[5] Whereas in the four studies that used the St. George's Respiratory Questionnaire there was a sizeable difference between the intervention and control groups (mean (95% CI): -7 (-12 to -3)), the mean between-group difference of the Chronic Respiratory Questionnaire dyspnoea (CRQ-D) and fatigue domains (CRQ-F) did not reach the minimum clinically important difference (MCID) in three other studies.[5] Only one of the eight studies measured muscle strength but results were reported descriptively with no statistical testing.[5] Furthermore, only one of the eight studies would fulfil the BTS guidelines definition of PR,[1] with seven studies not offering any education component.[5]

To our knowledge, no trial has compared supervised centre-based PR using minimal and specialist equipment. A previous National Institute for Health Research Health Technology Assessment-funded randomised 2x2 trial compared PR undertaken in community venues with PR undertaken in a hospital venue, in 240 patients with COPD.[6] Participants were block randomized (hospital n=129; community n=111). Both groups received twice-weekly PR for six weeks with the exercise training protocol identical in both venues. Importantly, neither community nor hospital sites had access to specialist exercise equipment. Patients in both groups improved their walking distance, exceeding the MCID of the endurance

For peer review only - http://bmjopen.bmj.com/site/about/guidelines.xhtml

shuttle walk test, and there were similar improvements in health-related quality of life.[6] Although the results support the conclusion that the efficacy of PR-min is not influenced by hospital or community locations, this study did not address whether PR-min is non-inferior to 'gold standard' PR-gymOur group recently conducted an observational cohort study to compare if an eight-week supervised, centre-based PR-min programme was non-inferior to PR-gym in terms of core PR outcomes in patients with COPD.[7] Using propensity score matching, 318 consecutive patients undergoing PR-min were compared 1:1 with a control group of 318 patients who underwent PR-gym. Similar short-term improvements in exercise capacity and health-related quality of life were observed in both groups (mean (95% CI) difference: ISW: 3 (-16 to 9) metres; CRQ-total: 0.9 (-2.7 to 4.5)). Furthermore, the 95% confidence interval between-group differences for these outcomes did not cross the pre-defined non-inferiority margins. These data suggest that PR-min is non-inferior to PR-gym with regards to short term improvements in exercise capacity and health-related quality of life. However, this study was not randomised, only included short term outcomes and no attempt was made to estimate costs. A randomised controlled trial with more varied outcome measures (such as lower limb muscle strength), longer follow-up and a health economic analysis, is required to confirm these findings.

The aims of the research are to determine whether an eight-week outpatient supervised PR-min programme is non-inferior to a standard eight-week outpatient supervised PR-gym programme in terms of health benefits for patients with chronic respiratory disease.

The primary objective is to determine whether PR-min is non-inferior to PR-gym regarding change in exercise capacity measured using ISW[8] distance from baseline (Visit 1) to post-PR assessment at eight weeks (Visit 2).

The secondary objectives are to: 1) determine whether PR-min is non-inferior to PR-gym regarding changes breathlessness (CRQ–D), health-related quality of life (CRQ) and quadriceps strength (isometric quadriceps maximum voluntary contraction) from baseline (Visit 1) to post-PR assessment at eight weeks (Visit 2) and

For peer review only - http://bmjopen.bmj.com/site/about/guidelines.xhtml

from baseline (Visit 1) to 12 months after the baseline assessment (Visit 3); 2) evaluate the trial process by recording the participant recruitment and retention, participant uptake of PR, PR attendance, PR completion, reasons for PR non-completion and participant satisfaction in each arm of the study, at the appropriate stage of the trial; 3) estimate the cost and cost-effectiveness during the trial period.

Methods and analysis:

Ethical approval

This study has been approved by the Health Research Authority and London Camden and Kings Cross Research Ethics Committee (reference 18/LO/0315). All participants will provide written informed consent.

Study design

This is a parallel, two-group, assessor- and statistician-blinded, non-inferiority, randomised trial. Participants will be randomised at the individual level with a 1:1 allocation to either PR-min or PR-gym. Both interventions will comprise two supervised sessions per week for eight weeks delivered by the same team. Outcome measures will be recorded at baseline assessment for PR (Visit 1), following PR at eight weeks (Visit 2) and at 12 months after Visit 1 (Visit 3). The study schema is outlined in figure 1.

Study population

Potential participants will be people referred to Harefield Pulmonary Rehabilitation Unit, Royal Brompton and Harefield Clinical Group, UK who meet the following inclusion criteria 1) adults >18 years of age; 2) physician diagnosis of stable chronic respiratory disease, typically COPD, interstitial lung disease, bronchiectasis, chronic asthma or chest wall disease; 3) referred for PR in line with BTS guidelines (i.e. ambulatory – can walk ≥5 metres, functional impairment related to breathlessness, typically Medical Research Council dyspnoea score ≥2); 4) able to communicate verbally and respond to questions in written

For peer review only - http://bmjopen.bmj.com/site/about/guidelines.xhtml

English. Potential participants will be excluded if they have 1) any contraindication to moderate intensity physical exercise e.g. unstable cardiovascular disease; 2) progressive cancer or neurological disorder with an expected life expectancy less than 12 months; 3) completed PR within previous 12 months and/ or 4) are unable to provide informed consent.

Consent

Informed consent will be obtained by the nominated researchers as recorded in the Sponsor's Delegation of Responsibilities Log. All individuals taking informed consent will have received consent training. All participants will provide written consent. Consent to enter this study will be obtained after a full account has been provided of its nature, purpose, risks, burdens and potential benefits, and the patient has had the opportunity to deliberate. The patient will be allowed to specify the time they wish to spend deliberating, usually up to 24 hours. The Investigator or designee will explain that the patients are under no obligation to enter the study and that they can withdraw at any time during the study, without having to give a reason. At each visit, the participant's willingness to continue in the study will be ascertained and documented in the research notes. A copy of the consent form is in the online supplement.

Screening and recruitment

Following clinical referral, but prior to pre-PR assessment, potential participants will be screened by a member of the PR team for eligibility to the study. The potential participant will be approached by telephone and provided with a participant information sheet. A member of the research team will obtain informed consent following the pre-PR assessment.

Randomisation, allocation and blinding procedures

Consenting participants will be randomised at the individual level with a 1:1 allocation, using an independent web-based system provided by the United Kingdom Clinical Research Collaboration registered King's Clinical Trials Unit (KCTU), to receive either "usual care" (PR-gym) or intervention (PR-min).

For peer review only - http://bmjopen.bmj.com/site/about/guidelines.xhtml

For peer review only

Randomisation by minimisation will ensure that participant characteristics are balanced between the groups with respect to previous completion of PR (yes/no), multiple deprivation index (most deprived quintile of index: yes/no[9]) and frailty status (Short Physical Performance Battery score < 10/≥10[10]). A proportion of patients will be entered initially using simple randomisation in order to create a level of initial imbalance and the minimisation algorithm will maintain a level of randomness in order to preserve pre-randomisation allocation concealment. Once randomised, the system will automatically generate a full audit trail of the process and send emails to relevant investigators in a blinded or unblinded format, depending on their role. For each arm, participants will be provided with a choice of three sites in northwest London to undertake PR.

Owing to the nature of the interventions, participants and providers of the intervention will not be blinded. However post-rehabilitation assessments will be performed by a researcher blinded to group allocation and not involved in the delivery of either intervention arm. The trial statistician will also be blinded to group allocation.

Study intervention

PR-gym (control group): The control intervention will be current gold-standard clinical practice. PR-gym will comprise an eight-week outpatient exercise and multi-disciplinary self-management education programme, with two supervised and at least one additional home session each week, and delivered according to the BTS Quality Standards for PR.[11] Supervising staff will comprise specialist respiratory therapists with a minimum of two years' experience in PR. Available equipment will include treadmills, cycle ergometers, cross-trainers, specialist lower limb resistance equipment (e.g. leg press, knee extension). Each supervised session will consist of one hour of exercise (at least 30 minutes aerobic exercise) and 45 minutes of education.

Initial walking speed prescription on the treadmill will be 80% of predicted peak oxygen consumption based on baseline ISW performance[8], whilst initial endurance cycling will be initially set to achieve level 3

For peer review only - http://bmjopen.bmj.com/site/about/guidelines.xhtml

to 4 on the Borg CR-10 Dyspnoea Scale with the aim of patients completing ten minutes of continuous training. Lower limb resistance training will comprise two sets of ten leg press repetitions on specialist resistance equipment performed with an initial training load of 60% one-repetition maximum. Similarly, two sets of ten bilateral knee extension repetitions will be performed on specialist resistance equipment at an initial training load of 60% one-repetition maximum. This will be supplemented with sit-to-stand sets, plus knee lifts/extension and hip abduction with appropriate ankle weights up to 10kg. Upper limb resistance training will comprise biceps curls, shoulder press and upright row with free weights or Therabands™ (red to black). Exercise training will be individualised and regularly progressed (duration and/or intensity) according to standard operating procedures, with targets reviewed at each session. Education will be delivered by a multi-disciplinary team with topics chosen to develop patients' understanding and holistic management of their disease. Further detail on the education programme and safety measures for the rehabilitation programme are provided in the online supplement.

PR-min (Intervention group): PR-min will also comprise an eight-week outpatient exercise and multidisciplinary self-management education programme, with two supervised and at least one additional home session each week, and delivered according to the BTS Quality Standards for PR.[11] As for PR-gym, supervising staff will comprise specialist respiratory therapists with at least two years' experience of in PR. There will be no access to treadmills, cycle ergometers, or specialist resistance equipment. Available exercise equipment will include walking circuit, portable steppers, portable pedals, hand and ankle weights (up to 5kg), and Therabands™ (red to black). Each supervised session will consist of one hour of exercise (at least 30 minutes aerobic exercise) and 45 minutes of education.

Initial walking speed prescription will be 80% of predicted peak oxygen consumption based on baseline ISW performance. Participants will be provided with a stopwatch and given time targets to complete a walking course of known distance. Although the resistance of the portable steppers and pedals can be manually adjusted, this cannot be objectively quantified. Initial prescription will be set at "level 1" but individually adjusted to find an intensity where patients can complete ten minutes of continuous training

For peer review only - http://bmjopen.bmj.com/site/about/guidelines.xhtml

with a target modified Borg breathless score of 3-4 and a Borg rating of Perceived Exertion of 13-15 (on a scale of 6-20). Resistance training will include functional activities such as sit-to-stand and step-ups as well as Theraband™ based exercises such as sitting knee extension, leg press and hip flexion as well as standing hip extension, squats, chest press and lateral raise. Progression will be through the use of hand/ankle weights and increasing resistant Therabands™ (from red to black). Exercise training will be individualised and regularly progressed (either in duration or intensity) with targets reviewed at each supervised session. Education will be delivered by a multi-disciplinary team as per PR-gym (online supplement). Information on safety measures for the rehabilitation programme is provided in the online supplement.

<ins>Study outcome measures</ins>

A list of the scheduled outcomes is outlined in table 1 and described in detail in the online supplement.

For peer review only

For peer review only - http://bmjopen.bmj.com/site/about/guidelines.xhtml

Table 1. Study outcome measures

	Visit 1	Visit 2	Visit 3
Primary outcome measure: Exercise capacity (Incremental Shuttle Walk Test)	X	X	X
Spirometry	X		X
Frailty (Short Physical Performance Battery)	X	X	X
Dyspnoea (Chronic Respiratory Questionnaire – Dyspnoea domain)	X	X	X
Health-related quality of life (Chronic Respiratory Questionnaire)	X	X	X
Muscle strength (Isometric Quadriceps Maximum Voluntary Contraction)	X	X	X
Patient satisfaction (Global Rating of Change Questionnaire)		X	
Health-economic evaluation (Modified Client Service Receipt Inventory Scale, Euro-Qol-5 Dimensions-5 Levels, NHS Digital - after visit 3 only)	X	X	X
Safety and trial process evaluation	X	X	X

Sample size calculation

Previous audits of Harefield PR Unit have shown that in participants undergoing PR-gym achieve a mean (standard deviation) change in ISW of 58 (67) metres. The null hypothesis is that PR-min is inferior to the standard treatment PR-gym. The alternative hypothesis is that PR-min is not inferior to PR-gym. The non-inferiority margin will be defined as half the known MCID using the fixed-margin method with a preserved effect of 50% as recommended by previous guidance, including from the United States Food and Drug Administration.[12 13] The MCID of the ISW is 47.5 metres,[14] and therefore 24 metres will be considered the non-inferiority margin. If there is truly no difference between PR-min and PR-gym, then a minimum of 246 patients (123 in each group) is required to be 80% sure that the lower limit of a one-sided 97.5% CI (or equivalently a 95% two-sided CI) will be above the non-inferiority limit of -24 metres. Based on audit data, we anticipate 32% drop out from PR (12% from assessment to starting PR, and 20% from starting PR to completing PR). Taking into account drop-out, the originalminimum sample size required for analysis was 362 patients (181 patients per group). However, owing to the impact of the COVID-19 pandemic on the

For peer review only - http://bmjopen.bmj.com/site/about/guidelines.xhtml

study (described in the Amendment section), the study sample size was increased by a further 74 to 436 (218 patients per group).

Statistical analysis

For baseline data, continuous and categorical variables will be summarized with descriptive statistics. No significance testing will be carried out. The outcome measures will be described by trial arm and at visits 1, 2 and 3 using descriptive statistics.

The main statistical analyses will estimate the difference in mean primary and secondary outcomes between patients randomised to PR-gym and PR-min by intention to treat principle from visit 1 to visit 2 (eight weeks following visit 1) and visit 1 to visit 3 (12 months following visit 1). Group difference estimates and associated one-sided 97.5% confidence intervals will be reported. The group differences will be compared using one-sided two independent sample t-test or non-parametric equivalent. The significance level is set at one-sided significance level of 0.025.

Regarding missing data, the number of baseline variables with complete data will be reported and the primary and secondary outcomes will be analysed as per the recommendations of White et al.[15] Missing post-randomisation assessments will be dealt with using an appropriate method of imputation, depending on the missing mechanism.

Adherence is defined as the number of supervised sessions the participant attends irrespective of the assigned intervention (maximum 16). This will be recorded in both intervention arms. As a dichotomous indicator with eight as the cut off. The following variables will be recorded in a 3x2 table according to allocated intervention: 1) compliers always receive the allocated treatment; 2) complete defiers do the opposite of the allocated treatment i.e. attend no sessions in allocated treatment; 3) partial defiers are all others not falling in the above groups. Dropout cause will be recorded using the MORECARE classification of reason for attrition.[16]

For peer review only - http://bmjopen.bmj.com/site/about/guidelines.xhtml

In addition to the primary intention-to-treat analysis, the effect of actually receiving treatment as defined in the protocol will also be estimated. If non-compliance rate with PR sessions is >10%, a Complier-Average Causal-Effect (CACE) will be estimated.[17] The following sensitivity analyses are planned: 1) per protocol; 2) including only those in the upper quartile for baseline ISW; 3) complete case; 4) complier Average Causal Effects (CACE) analysis[17] and 5) primary diagnosis of COPD; 6) a generalised estimating equation-based analysis to estimate the treatment effects, adjusting for imbalance if the group difference in the percentage of smokers, participants age <70 years, or females is greater than 20% at baseline.

Data collection, management and monitoring

A web based electronic data capture (EDC) system has been designed using the InferMed Macro 4 system and will be maintained by KCTU for the duration of the project. Source data will be entered by recruiting site staff, typically within one week of data collection by authorised staff onto the EDC. A full audit trial of data entry and any subsequent changes to entered data will be automatically date and time stamped, alongside information about the user making the entry/changes within the system.

The primary investigator (WM) will take overall responsibility for the conduct of MISTER. The trial co-ordinator (CMN) will supervise the day-to-day operation of the project and is responsible for ensuring that Good Clinical Practice guidelines are followed. An unblinded member of the MISTER research team (JAW) will monitor the data and review a random sample of 10% of completed case report forms against clinical records. Monitoring will ensure protocol compliance, proper study management and timely completion of study procedures.

Monitoring and auditing

A Data Monitoring and Ethics Committee (DMEC) has been appointed. The members of this committee are not members of the applicants' or sponsors' institution and include an academic Consultant Respiratory Physician and an independent statistician.

For peer review only - http://bmjopen.bmj.com/site/about/guidelines.xhtml

The requirement for study monitoring or audit will be based on the Sponsor's Research Office risk assessment procedure and applicable standard operating procedures. It is the responsibility of the Research Office to determine the monitoring risk assessment and explain the rationale to the study research team.

Patient and public involvement (PPI)

The concept of the research project arose directly from PPI, and the development of this project has included PPI throughout each stage. We have a named PPI co-applicant who will continue to be closely involved in the design and management of the research through membership of the Trial Steering Group that will meet at the beginning of the research period, and then every six months throughout the data collection/analysis and dissemination period. This PPI member will be joined by a second representative and will meet the project manager at regular intervals throughout the study. The PPI group will provide input into written material for patients, how results data are presented, particularly to lay audiences and will have a role in dissemination of research findings.

Safety monitoring

A Serious Adverse Event (SAE) is defined as an untoward occurrence that a) results in death; b) is life-threatening; c) requires hospitalization or prolongation of existing hospitalization; d) results in persistent or significant disability or incapacity or e) is otherwise considered medically significant by the investigator.

The Investigator and research team are responsible for reporting events to the Research Office immediately and/or within 24 hours of becoming aware of the event. An SAE occurring to a research participant will be reported to the Research Ethics Committee that gave a favorable opinion of the study.

Amendment

Owing to the COVID-19 pandemic the study was suspended for 13 months between February 2020 and 2021, and, at the date of study suspension, there were 74 recruited and randomised participants waiting to start pulmonary rehabilitation but were unable to receive either intervention due to the pandemic. Accordingly,

For peer review only - http://bmjopen.bmj.com/site/about/guidelines.xhtml

a substantial amendment to increase the sample size from 362 to 436 and extend the recruitment and follow-up periods by one year to 31st March 2022 and 2023 respectively was submitted to the funders (National Institute for Health Research), the Health Research Authority and research ethics committee on 5th February 2021. These amendments were approved on 24th February 2021.

Should further substantial amendments be made, a notice of amendment will be submitted to the Health Research Authority for consideration.

Study timelines

Participant recruitment started on 15th October 2018 and is expected to end on 31st March 2022. Visit 2 assessments started approximately 8 weeks after Visit 1 and Visit 3 assessments 12 months after Visit 1. The final Visit 2 assessment is expected to take place on 31st May 2023. The end date for the study, representing the final Visit 3 will be 31st March 2023.

Dissemination:

We will utilise a broad strategy to maximise dissemination of our findings: 1) sharing of scientific findings via open-access publication in high impact journals and presentation at international meetings; 2) plain English summaries of findings for public bodies and web-sites (e.g. NIHR, CLARHC, British Lung Foundation, patient.co.uk) to communicate evidence through a user-friendly interface; 3) online pages about the project on websites of contributing organisations (Guy's and St Thomas' NHS Foundation Trust, Cicely Saunders Institute, King's College London); 4) social media (e.g. Twitter); 5) public engagement via talks with service user groups and open public events so patients and their caregivers can learn about the research; 6) direct sharing of findings with public bodies (e.g. National Horizon Scanning Research and Intelligence Centre) and policy makers to whom the applicants have direct access (e.g. BTS Pulmonary Rehabilitation Advisory Group, European Respiratory Society (ERS), American Thoracic Society (ATS),

For peer review only

For peer review only - http://bmjopen.bmj.com/site/about/guidelines.xhtml

Society for Research in Rehabilitation) to facilitate uptake of findings into research strategy and policy; 7) education of commissioners to influence future NHS service delivery.

Discussion:

PR is a core strategy in the management for COPD and other chronic respiratory disorders.[1] The majority of evidence to support PR has come from trials conducted in locations that utilise specialist exercise equipment.[2] Data from a national audit indicate that many PR programmes in England and Wales do not have access to specialist exercise equipment with exercise training completed using simple equipment.[3] There is a paucity of robust literature examining the efficacy of PR-min and to our knowledge, there have been no trials comparing supervised centre-based PR-min and PR-gym. One observational study demonstrated that PR-min is non-inferior to PR-gym in terms of exercise capacity and health-related quality of life but indicated that further investigation using a randomised controlled trial is required.[7] Therefore, this study aims to bridge the gap in knowledge by identifying whether PR-min is non-inferior to PR-gym regarding change in core PR outcome measures. This is an important and current health service question given the increasing disparity between supply and demand of PR that has implications for the delivery and future development of this programme at national and international levels.

The strengths of this study include the design: a parallel, two-group, assessor- and statistician-blinded, non-inferiority randomised trial which is the appropriate design to assess the primary and secondary outcome measures. The intervention, PR, is delivered according to national guidelines and the population of interest is people with chronic respiratory disease, both of which mean the results will be generalisable. In addition, study involves a 12-month follow-up period (from Visit 1) to evaluate the long-term effect of the intervention and a health-economic analysis which aims to understand the potential cost-effectiveness

For peer review only - http://bmjopen.bmj.com/site/about/guidelines.xhtml

of the trial which may have implications for the NHS and Clinical Commissioning Groups. A limitation of the study is that it is a single-centre study based in northwest London which may mean that the findings may not be generalisable to other populations in different locations.

Acknowledgements

We would like to acknowledge Joanna Kelly, Evangelos Georgio, Janice Jimenez and their teams in the Kings Clinical Trials Unit data centre who developed and will maintain the randomisation and Elsevier MACRO database systems for the trial. We would also like to acknowledge Ms Peihan Yu, statistician at Kings College London, for her help developing the study protocol. We would like to express our gratitude to the Harefield Pulmonary Rehabilitation Unit, Royal Brompton and Harefield Clinical Group, Guy's and St Thomas' NHS Foundation Trust who will help with participant identification and delivery of the pulmonary rehabilitation programme.

Figure legend

Figure 1. Study schema.

For peer review only

Full references

1. Bolton CE, Blakey JD, Morgan MD. The British Thoracic Society guideline on pulmonary rehabilitation in adults: your opinion is noted. *Thorax* 2014;69(4):388-9. doi: 10.1136/thoraxjnl-2013-204754 [published Online First: 2014/01/17]
2. McCarthy B, Casey D, Devane D, et al. Pulmonary rehabilitation for chronic obstructive pulmonary disease. *The Cochrane database of systematic reviews* 2015;2 doi: 10.1002/14651858.CD003793.pub3 [published Online First: 2015/02/24]
3. RCP B. National COPD Audit Programme. Pulmonary Rehabilitation: Steps to Breathe Better 2016 [Available from: file:///C:/Users/cn805/Downloads/Nat%20COPD%20PR%20Clinical%20Audit%202015%20Full%20report_0.pdf accessed 4th December 2017.
4. Steiner M, McMillan V, Lowe D, et al. Pulmonary rehabilitation: an exercise in improvement. *National chronic obstructive pulmonary disease (COPD) audit programme: clinical and organisational audits of pulmonary rehabilitation services in England and Wales* 2017
5. Alison JA, McKeough ZJ. Pulmonary rehabilitation for COPD: are programs with minimal exercise equipment effective? *J Thorac Dis* 2014;6(11):1606.
6. Waterhouse J, Walters S, Oluboyede Y, et al. A randomised 2 x 2 trial of community versus hospital pulmonary rehabilitation, followed by telephone or conventional follow-up. *Health technology assessment (Winchester, England)* 2010;14(6):i-v, vii-xi, 1-140.
7. Patel S, Palmer MD, Nolan CM, et al. Supervised pulmonary rehabilitation using minimal or specialist exercise equipment in COPD: a propensity-matched analysis. *Thorax* 2020 doi: 10.1136/thoraxjnl-2020-215281 [published Online First: 2020/11/03]
8. Singh SJ, Morgan MD, Scott S, et al. Development of a shuttle walking test of disability in patients with chronic airways obstruction. *Thorax* 1992;47(12):1019-24.
9. Ministry for Housing Communities and Local Government. English Indices of Deprivation 2015 2015 [Available from: http://imd-by-postcode.opendatacommunities.org/imd/2015 accessed 3rd November 2020.
10. European Medicines Agency. Reflection paper on physical frailty: instruments for baseline characterisation of older populations in clinical

trials 2018 [Available from: https://www.ema.europa.eu/en/documents/scientific-guideline/reflection-paper-physical-frailty-instruments-baseline-characterisation-older-populations-clinical_en.pdf accessed 22nd October 2020.
11. BTS. Quality Standards for Pulmonary Rehabilitation. *British Thoracic Society Reports* 2014;6(2)
12. Piaggio G, Elbourne DR, Pocock SJ, et al. Reporting of noninferiority and equivalence randomized trials: extension of the CONSORT 2010 statement. *Jama* 2012;308(24):2594-604.
13. Wangge G, Putzeist M, Knol MJ, et al. Regulatory scientific advice on non-inferiority drug trials. *PloS one* 2013;8(9):e74818.
14. Singh SJ, Jones PW, Evans R, et al. Minimum clinically important improvement for the incremental shuttle walking test. *Thorax* 2008;63(9):775-7.
15. White IR, Horton NJ, Carpenter J, et al. Strategy for intention to treat analysis in randomised trials with missing outcome data. *Bmj* 2011;342:d40.
16. Higginson IJ, Evans CJ, Grande G, et al. Evaluating complex interventions in end of life care: the MORECare statement on good practice generated by a synthesis of transparent expert consultations and systematic reviews. *BMC medicine* 2013;11(1):111.
17. Jo B. Statistical power in randomized intervention studies with noncompliance. *Psychological Methods* 2002;7(2):178.

For peer review only - http://bmjopen.bmj.com/site/about/guidelines.xhtml

Authors' contributions: CMN, MM, WG and WD-CM: Concept and design of the study; CMN, JW, SP, REB, OP: Data collection and entry; CMN, MM, GW and WD-CM: Drafted the manuscript; WG, RW, MM, CMN and WD-CM: Drafted the statistical analysis plan. All authors reviewed the final draft of the manuscript.

Funding statement: This work is supported by National Institute for Health Research grant number PB-PG-0816-20022.

Competing interests statement

JAW, SP, REB, OP, RW, WG have no competing interests

CMN reports fees from Novartis outside of this research.

MM consultancy fees from Fresenius Kabi and Helsinn, grants from National Institute for Health Research, grants from British Lung Foundation, outside the submitted work.

WD-CM reports personal fees from Jazz Pharmaceuticals, personal fees from Mundipharma, personal fees from Novartis, grants from Pfizer, non-financial support from GSK, grants from National Institute for Health Research, grants from British Lung Foundation, outside the submitted work.

Word Count: 4128

For peer review only

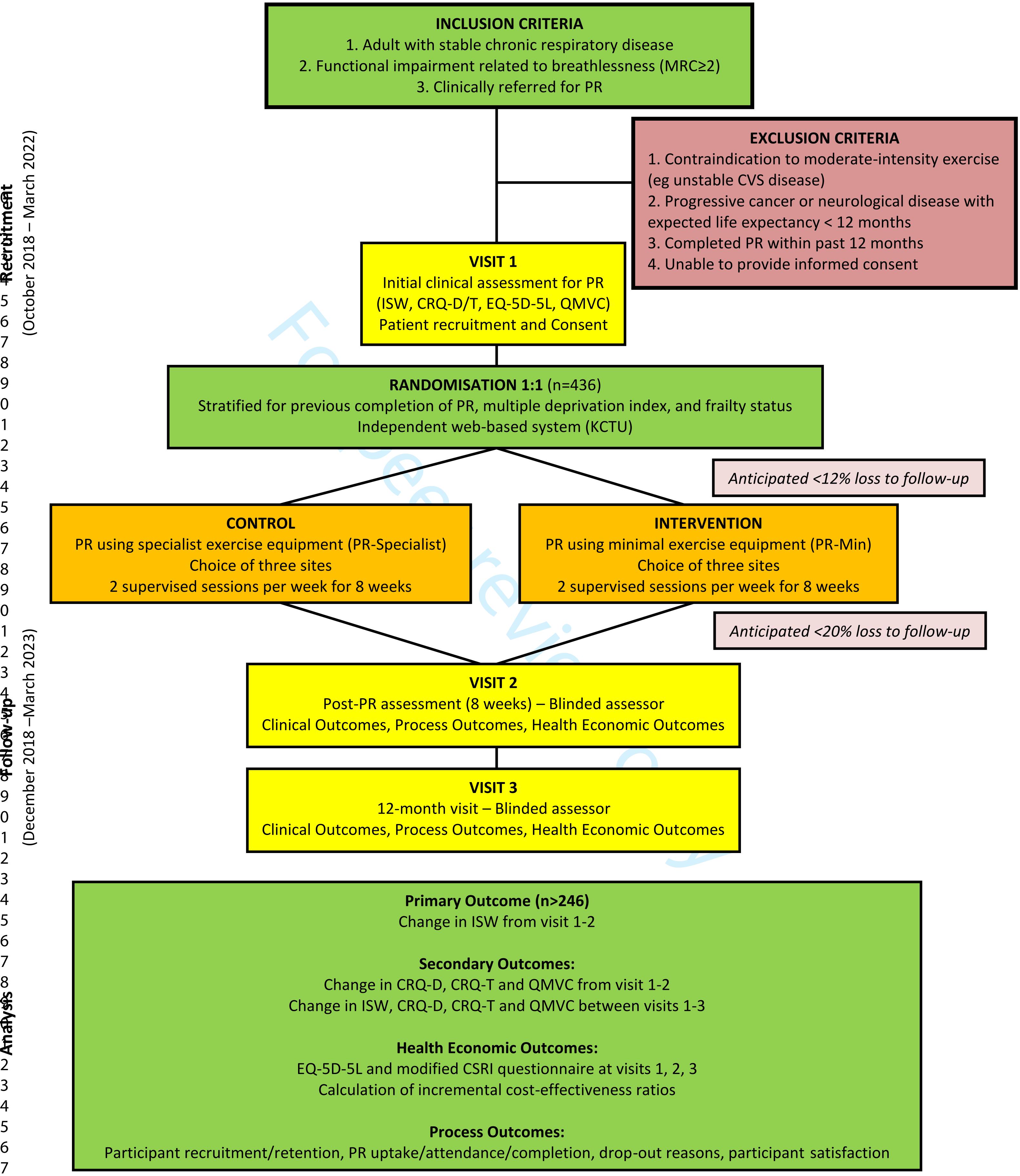

For peer review only - http://bmjopen.bmj.com/site/about/guidelines.xhtml

Minimal versus specialist equipment in the delivery of pulmonary rehabilitation : Protocol for a non-inferiority randomised controlled trial

Online supplement

Consent: Informed consent form

For peer review only

PARTICIPANT CONSENT FORM

MInimal versus SpecialisT Equipment in the delivery of pulmonary Rehabilitation (MISTER): a randomised controlled trial

IRAS Reference: 241564

Name of Researcher: ....

Participant study ID number: ....

Please initial the boxes

1. I confirm that I have read and understand the participant information leaflet (version **4** dated **02/02/2021**) for the above study. I have had the opportunity to consider the information, ask questions and have had these answered satisfactorily. ☐

2. I understand that my participation is voluntary and that I am free to withdraw at any time without giving any reason, although data collected until that point may be used for research purposes with my agreement, without my medical care or legal rights being affected. ☐

3. I understand that sections of my medical notes relating to the study may be looked at by regulatory bodies conducting inspections which may include members of staff within the NHS Trust who are outside of the immediate clinical and research teams. I give permission for these individuals to have access to my records. ☐

4. a) I agree that the information held and maintained by NHS Digital and other NHS bodies will be used to provide information about my healthcare use, and in the case of my death, information about this. ☐

   b) I consent to share identifiable details (name, NHS number, date of birth, post-code) with NHS Digital. ☐

5. I consent to allowing my postcode to be used to calculate the Multiple Deprivation Index. ☐

6. I agree to my GP being informed of my participation in the study. ☐

7. I agree to take part in the above study. ☐

8. I wish to receive feedback on the study results when available. ☐

Name of Patient	Date	Signature
Name of Person taking consent (If different from researcher)	Date	Signature
Researcher	Date	Signature

When completed: 1 for patient; 1 (original) for researcher site file; 1 to be kept in medical notes

Study intervention

Home-exercise manual and study standard operating procedures: These can be provided on application to the Royal Brompton and Harefield Hospitals.

Education component of pulmonary rehabilitation

A multidisciplinary team, including physiotherapists, psychologists, dieticians, nurses, doctors, occupational therapists, dieticians, social workers, speech and language therapists and expert patients, delivered the education sessions. The will aim to develop patients' understanding and holistic management of their disease, and topics included pacing strategies, breathing control exercises, physical activity and exercise, medication use, diet, smoking cessation, management of anxiety and low mood, managing infections through early recognition, rescue medication, appropriate general practice/hospital presentation and loving relationships. The sessions will take place twice per week for 45 minutes and patients will receive a booklet of the topics covered in these sessions.

Safety measures during PR-min and PR-gym rehabilitation programmes

Safety measures for both types of programme:

- The programme shall be delivered at a suitable time and in easily accessible buildings and locations with adequate parking, good transport links and suitable for people with disabilities.
- A minimum of two members of staff are required to supervise group exercise classes and education sessions – one of whom must be a registered allied health professional.
- There will be a minimum of two members of staff supervising group exercise classes and education sessions in community settings. There is an absolute minimum of one staff to eight people for supervised group exercise. However, the ratio may increase to one staff to four people depending on the complexity and severity of the medical problems, and will be at the judgement of the responsible member of the senior clinical staff
- There should be no more than two ambulatory or long-term oxygen users in any one exercise class.
- Prior to each session a risk and suitability assessment of the venue must be undertaken.

For peer review only - http://bmjopen.bmj.com/site/about/guidelines.xhtml

- Unwell patients are managed using the emergency care protocol. In short, if urgent help is required in a community setting, call an ambulance, and in a hospital setting, call the crash team.
- Contraindications to exercise shall be screened at the pulmonary rehabilitation assessmen.
- Patients shall stop exercising if $Sp0_2$ <80% and if they feel unwell e.g. chest pain, nausea or if a member of staff feels it is necessary.
- The following monitoring equipment is required: pulse oximeters, blood pressure monitor
- The following emergency equipment is required:
    - Community-based programmes: portable defibrillator and bag-valve mask
    - Hospital-based programmes: crash trolley
    - Portable oxygen, tubing, face-mask
    - Glucose kit
    - Bio-hazard spill kit
    - First aid kit

Study outcome measures

Spirometry: Visit 1 and visit 3

All participants will undergo spirometry performing the Forced Vital Capacity (FVC) / Forced Expiratory Volume in one second (FEV_1) manoeuvre as described in Appendix 1, using an EasyOne™ diagnostic spirometer. As a minimum, three technically acceptable tests must be performed and must meet the Association of Respiratory Technology and Physiology reproducibility criteria.[11]. Results obtained will include FVC, FEV_1 and the FEV_1/FVC ratio. Percent predicted reference equations from the European Respiratory Society/European Steel and Coal Society were used.[12]

Frailty: Visit 1, visit 2 and visit 3

All participants will perform the Short Physical Performance Battery (SPPB) which is a simple test of lower limb functional performance and a marker of frailty.[13] It comprises an assessment of standing balance, usual walking speed

For peer review only - http://bmjopen.bmj.com/site/about/guidelines.xhtml

and ability to stand from a chair. The SPPB is scored out of 12 with a higher score indicating better functional performance. A score of <10 indicates a frail status and this cut-off will be used in the randomisation procedure.

Exercise capacity: Visit 1, visit 2 and visit 3

The ISW is an incremental, externally paced, field walking test that involves participants walking around a 10 metre course in time to a series of progressively faster beeps played from a CD player. At visit 1, two tests (a practice test and then the formal test) will be undertaken with at least 30 minutes rest between tests.[14] This is in accordance with international technical standards. Measures of breathlessness, oxygen saturation levels and heart rate will be recorded before and after the test. The distance completed in metres will also be recorded.

Dyspnoea: Visit 1, visit 2 and visit 3

Dyspnoea will be measured using the CRQ-D.[15] The dyspnoea domain allows the patients to choose five activities that have been limited by shortness of breath in the past two weeks.

Health-related quality of life: Visit 1, visit 2 and visit 3

Health-related quality of life will be measured using the CRQ.[15] This 20 item questionnaire, is responsive to PR, and contains four domains; dyspnea (described above), fatigue, emotional function and mastery, and a total score. Each item is scored on a seven point Likert scale, with a lower score indicating a higher symptom burden.

Muscle strength: Visit 1, visit 2 and visit 3

Isometric quadriceps maximal voluntary contraction of the dominant leg will be measured using specially designed chair and strain gauge. This test involves the participant pushing against an ankle strap with the knee positioned at 90^0.[16] A warm-up and six efforts will be performed.

For peer review only - http://bmjopen.bmj.com/site/about/guidelines.xhtml

Patient satisfaction: Visit 2

To measure patient satisfaction, participants will rate their response to the following question *"How do you feel your overall condition has changed after rehabilitation?"* on a five point Global Rating of Change Questionnaire. The scale ranges from *"1: I feel much better"* to *"5: I feel much worse"*.

Health economic evaluation: Visit 1, visit 2 and visit 3

- The Modified Client Service Receipt Inventory questionnaire will be used to record information on health care resource, medicine and equipment use, informal care provided by family members, time off work and costs borne by the patient and family relating to the chronic lung disease.
- The EQ5D5L is a generic measure of health status that comprises a visual analogue scale and five-item questionnaire with the following domains: mobility; self-care; usual activities; pain/discomfort and anxiety/depression.[17]
- After visit 3, data will be obtained from NHS Digital regarding health resource utilization between visit 1 and visit 3. This will include information on elective and non-elective hospital admissions (e.g. reason for admission; type of ward; treatment received; length of stay (including date of admission and discharge); location of discharge) as well as outpatient healthcare contacts (e.g. type of appointment, number of contacts, treatment received) and mortality (e.g. date of death, reason for death, place of death).

Safety and trial process evaluation: Visit 1 and visit 2

- Safety will be assessed in real-time using adverse event reporting.
- PR uptake, adherence and completion will be assessed objectively through PR attendance registers and training records. Reasons for non-completion of PR will be assessed objectively through PR records.

For peer review only - http://bmjopen.bmj.com/site/about/guidelines.xhtml

SPIRIT

STANDARD PROTOCOL ITEMS: RECOMMENDATIONS FOR INTERVENTIONAL TRIALS

SPIRIT 2013 Checklist: Recommended items to address in a clinical trial protocol and related documents*

Section/item	Item No	Description
**Administrative information**		
Title	1	Descriptive title identifying the study design, population, interventions, and, if applicable, trial acronym
Trial registration	2a	Trial identifier and registry name. If not yet registered, name of intended registry
	2b	All items from the World Health Organization Trial Registration Data Set
Protocol version	3	Date and version identifier
Funding	4	Sources and types of financial, material, and other support
Roles and responsibilities	5a	Names, affiliations, and roles of protocol contributors
	5b	Name and contact information for the trial sponsor
	5c	Role of study sponsor and funders, if any, in study design; collection, management, analysis, and interpretation of data; writing of the report; and the decision to submit the report for publication, including whether they will have ultimate authority over any of these activities
	5d	Composition, roles, and responsibilities of the coordinating centre, steering committee, endpoint adjudication committee, data management team, and other individuals or groups overseeing the trial, if applicable (see Item 21a for data monitoring committee)
**Introduction**		
Background and rationale	6a	Description of research question and justification for undertaking the trial, including summary of relevant studies (published and unpublished) examining benefits and harms for each intervention
	6b	Explanation for choice of comparators
Objectives	7	Specific objectives or hypotheses
Trial design	8	Description of trial design including type of trial (eg, parallel group, crossover, factorial, single group), allocation ratio, and framework (eg, superiority, equivalence, noninferiority, exploratory)

For peer review only

Methods: Participants, interventions, and outcomes

Study setting	9	Description of study settings (eg, community clinic, academic hospital) and list of countries where data will be collected. Reference to where list of study sites can be obtained
Eligibility criteria	10	Inclusion and exclusion criteria for participants. If applicable, eligibility criteria for study centres and individuals who will perform the interventions (eg, surgeons, psychotherapists)
Interventions	11a	Interventions for each group with sufficient detail to allow replication, including how and when they will be administered
	11b	Criteria for discontinuing or modifying allocated interventions for a given trial participant (eg, drug dose change in response to harms, participant request, or improving/worsening disease)
	11c	Strategies to improve adherence to intervention protocols, and any procedures for monitoring adherence (eg, drug tablet return, laboratory tests)
	11d	Relevant concomitant care and interventions that are permitted or prohibited during the trial
Outcomes	12	Primary, secondary, and other outcomes, including the specific measurement variable (eg, systolic blood pressure), analysis metric (eg, change from baseline, final value, time to event), method of aggregation (eg, median, proportion), and time point for each outcome. Explanation of the clinical relevance of chosen efficacy and harm outcomes is strongly recommended
Participant timeline	13	Time schedule of enrolment, interventions (including any run-ins and washouts), assessments, and visits for participants. A schematic diagram is highly recommended (see Figure)
Sample size	14	Estimated number of participants needed to achieve study objectives and how it was determined, including clinical and statistical assumptions supporting any sample size calculations
Recruitment	15	Strategies for achieving adequate participant enrolment to reach target sample size

Methods: Assignment of interventions (for controlled trials)

Allocation:

Sequence generation	16a	Method of generating the allocation sequence (eg, computer-generated random numbers), and list of any factors for stratification. To reduce predictability of a random sequence, details of any planned restriction (eg, blocking) should be provided in a separate document that is unavailable to those who enrol participants or assign interventions

Allocation concealment mechanism	16b	Mechanism of implementing the allocation sequence (eg, central telephone; sequentially numbered, opaque, sealed envelopes), describing any steps to conceal the sequence until interventions are assigned
Implementation	16c	Who will generate the allocation sequence, who will enrol participants, and who will assign participants to interventions
Blinding (masking)	17a	Who will be blinded after assignment to interventions (eg, trial participants, care providers, outcome assessors, data analysts), and how
	17b	If blinded, circumstances under which unblinding is permissible, and procedure for revealing a participant's allocated intervention during the trial
**Methods: Data collection, management, and analysis**		
Data collection methods	18a	Plans for assessment and collection of outcome, baseline, and other trial data, including any related processes to promote data quality (eg, duplicate measurements, training of assessors) and a description of study instruments (eg, questionnaires, laboratory tests) along with their reliability and validity, if known. Reference to where data collection forms can be found, if not in the protocol
	18b	Plans to promote participant retention and complete follow-up, including list of any outcome data to be collected for participants who discontinue or deviate from intervention protocols
Data management	19	Plans for data entry, coding, security, and storage, including any related processes to promote data quality (eg, double data entry; range checks for data values). Reference to where details of data management procedures can be found, if not in the protocol
Statistical methods	20a	Statistical methods for analysing primary and secondary outcomes. Reference to where other details of the statistical analysis plan can be found, if not in the protocol
	20b	Methods for any additional analyses (eg, subgroup and adjusted analyses)
	20c	Definition of analysis population relating to protocol non-adherence (eg, as randomised analysis), and any statistical methods to handle missing data (eg, multiple imputation)
**Methods: Monitoring**		
Data monitoring	21a	Composition of data monitoring committee (DMC); summary of its role and reporting structure; statement of whether it is independent from the sponsor and competing interests; and reference to where further details about its charter can be found, if not in the protocol. Alternatively, an explanation of why a DMC is not needed

	21b	Description of any interim analyses and stopping guidelines, including who will have access to these interim results and make the final decision to terminate the trial
Harms	22	Plans for collecting, assessing, reporting, and managing solicited and spontaneously reported adverse events and other unintended effects of trial interventions or trial conduct
Auditing	23	Frequency and procedures for auditing trial conduct, if any, and whether the process will be independent from investigators and the sponsor

Ethics and dissemination

Research ethics approval	24	Plans for seeking research ethics committee/institutional review board (REC/IRB) approval
Protocol amendments	25	Plans for communicating important protocol modifications (eg, changes to eligibility criteria, outcomes, analyses) to relevant parties (eg, investigators, REC/IRBs, trial participants, trial registries, journals, regulators)
Consent or assent	26a	Who will obtain informed consent or assent from potential trial participants or authorised surrogates, and how (see Item 32)
	26b	Additional consent provisions for collection and use of participant data and biological specimens in ancillary studies, if applicable
Confidentiality	27	How personal information about potential and enrolled participants will be collected, shared, and maintained in order to protect confidentiality before, during, and after the trial
Declaration of interests	28	Financial and other competing interests for principal investigators for the overall trial and each study site
Access to data	29	Statement of who will have access to the final trial dataset, and disclosure of contractual agreements that limit such access for investigators
Ancillary and post-trial care	30	Provisions, if any, for ancillary and post-trial care, and for compensation to those who suffer harm from trial participation
Dissemination policy	31a	Plans for investigators and sponsor to communicate trial results to participants, healthcare professionals, the public, and other relevant groups (eg, via publication, reporting in results databases, or other data sharing arrangements), including any publication restrictions
	31b	Authorship eligibility guidelines and any intended use of professional writers
	31c	Plans, if any, for granting public access to the full protocol, participant-level dataset, and statistical code

For peer review only

Appendices

Informed consent materials	32	Model consent form and other related documentation given to participants and authorised surrogates
Biological specimens	33	Plans for collection, laboratory evaluation, and storage of biological specimens for genetic or molecular analysis in the current trial and for future use in ancillary studies, if applicable

*It is strongly recommended that this checklist be read in conjunction with the SPIRIT 2013 Explanation & Elaboration for important clarification on the items. Amendments to the protocol should be tracked and dated. The SPIRIT checklist is copyrighted by the SPIRIT Group under the Creative Commons "Attribution-NonCommercial-NoDerivs 3.0 Unported" license.

For peer review only

For peer review only - http://bmjopen.bmj.com/site/about/guidelines.xhtml
